# Contribution of Bursty Bulk Flows to the Global Dipolarization of the Magnetotail During an Isolated Substorm

**DOI:** 10.1029/2019JA026872

**Published:** 2019-11-13

**Authors:** V. G. Merkin, E. V. Panov, K. A. Sorathia, A. Y. Ukhorskiy

**Affiliations:** ^1^ The Johns Hopkins University Applied Physics Laboratory Laurel MD USA; ^2^ Space Research Institute Austrian Academy of Sciences Graz Austria

**Keywords:** global MHD, bursty bulk flow, dipolarization front, substorm, high resolution, multispacecraft

## Abstract

This paper addresses the question of the contribution of azimuthally localized flow channels and magnetic field dipolarizations embedded in them in the global dipolarization of the inner magnetosphere during substorms. We employ the high‐resolution Lyon‐Fedder‐Mobarry global magnetosphere magnetohydrodynamic model and simulate an isolated substorm event, which was observed by the geostationary satellites and by the Magnetospheric Multiscale spacecraft. The results of our simulations reveal that plasma sheet flow channels (bursty bulk flows, BBFs) and elementary dipolarizations (dipolarization fronts, DFs) occur in the growth phase of the substorm but are rare and do not penetrate to the geosynchronous orbit. The substorm onset is characterized by an abrupt increase in the occurrence and intensity of BBFs/DFs, which penetrate well earthward of the geosynchronous orbit during the expansion phase. These azimuthally localized structures are solely responsible for the global (in terms of the magnetic local time) dipolarization of the inner magnetosphere toward the end of the substorm expansion. Comparison with the geostationary satellites and Magnetospheric Multiscale data shows that the properties of the BBFs/DFs in the simulation are similar to those observed, which gives credence to the above results. Additionally, the simulation reveals many previously observed signatures of BBFs and DFs, including overshoots and oscillations around their equilibrium position, strong rebounds and vortical tailward flows, and the corresponding plasma sheet expansion and thinning.

## Key Points

- During substorm expansion all magnetic flux transport into the inner magnetosphere occurs via azimuthally localized earthward flows
- Substorm onset is characterized by an abrupt increase in the number of such flows penetrating to the geosynchronous orbit
- Properties of simulated bursty bulk flows/dipolarization fronts are similar to those observed including flux tube oscillations and rebounds

## 1 Introduction

Much of the plasma transport in the inner plasma sheet occurs by means of transient (∼10 min) flow enhancements known as bursty bulk flows (BBFs; e.g., Angelopoulos et al., 1992, 1994;Baumjohann et al., 1990). Since they typically carry significant northward magnetic field intensifications (Juusola et al., 2011; Nakamura et al., 2002; Ohtani et al., 2004; Runov et al., 2009), they also efficiently transport magnetic flux from the tail toward the inner magnetosphere (e.g., Liu et al., 2014). While individual dipolarizations have a limited azimuthal extent estimated at 1–3 Earth radii (*R*_E_; Angelopoulos et al., 1996; Liu et al., 2015; Nakamura et al., 2004; Sergeev et al., 1996), cumulatively, they may account for a large portion of the entire earthward flux transport in the plasma sheet (Angelopoulos et al., 1994).

Statistically, BBFs and associated magnetic field dipolarizations are observed in the inner plasma sheet more frequently with increasing distance from Earth (Angelopoulos et al., 1994; McPherron et al., 2011). However, dipolarizations do occur deeper in the inner magnetosphere (e.g., inside geosynchronous orbit; Ohtani et al., 2018), although their relationship with the fast flows and individual dipolarizations in the plasma sheet remains an open question (Ohtani et al., 2006; Ohtani et al., 2018; Sergeev et al., 2012; Takada et al., 2006). It has been suggested that the dipolarizations in the inner magnetosphere are caused by relatively rare BBFs that do penetrate to these smaller geocentric distances (Dubyagin et al., 2011; Sergeev et al., 2012)—a view consistent with the idea that BBFs correspond to propagating low flux tube entropy regions, or bubbles, that are widely considered as a solution to the so‐called pressure balance inconsistency (Erickson & Wolf, 1980; Pontius & Wolf, 1990). The magnetic field dipolarizations in the inner magnetosphere are often accompanied by injections of energetic particles, as revealed by many observational studies (e.g., Apatenkov et al., 2007;Mauk & McIlwain, 1974;Moore et al., 1981;Reeves et al., 1990), including more recent work using the Van Allen Probes (Gkioulidou et al., 2015; Liu et al., 2016; Motoba et al., 2018) and Van Allen Probes/Magnetospheric Multiscale (MMS) conjunction data (Turner et al., 2017). Thus, investigation of such dipolarizations and their relationship with BBFs in the plasma sheet is central both to quantification of the magnetic flux transport in the nightside magnetosphere and to understanding of the buildup of the ring current and radiation belts (e.g., Forsyth et al., 2016; Sandhu et al., 2018), as suggested by recent modeling efforts (Cramer et al., 2017; Sorathia et al., 2018; Ukhorskiy et al., 2018; Yang et al., 2011, 2015).

In this paper we consider the first of these problems that has direct bearing on the fundamental issue of magnetic flux circulation in the magnetosphere as originally posited by Dungey (1961). More specifically, we consider the contribution to a global‐scale dipolarization of the inner magnetosphere of individual azimuthally localized flows (BBFs) breaking in the near‐Earth plasma sheet (Baumjohann et al., 1999; Shiokawa et al., 1997), penetrating into the inner magnetosphere, and depositing there magnetic flux in the form of elementary dipolarizations (dipolarization fronts, DFs).

BBFs are observed in the plasma sheet during all substorm phases, but their occurrence frequency increases significantly during the expansion phase (e.g., Juusola et al., 2011). Thus, it is appropriate to ask the question, to what extent individual localized dipolarizations integrate into a large‐scale dipolarization of the inner magnetosphere characteristic of the substorms expansion (Kepko et al., 2015)? A closely associated question, whether the so‐called substorm current wedge (SCW) is composed of individual wedgelets, has received significant attention recently (Birn et al., 2011; Birn & Hesse, 2014b; Forsyth et al., 2014; Liu et al., 2015, 2018; Malykhin et al., 2018; Palin et al., 2016; Sergeev et al., 2000). Panov et al. (2016) used a fortuitous alignment of magnetospheric spacecraft and ground‐based magnetometers and imagers to infer that during a weak substorm (the *AE* index was about 100 nT) the substorm dipolarization could have been produced by a single BBF. However, the idea of SCW composition by wedgelets was first suggested to explain observations of multiple onsets of magnetic perturbations on the ground in the course of the same substorm (Baumjohann et al., 1981; Nakamura et al., 1994; Rostoker, 1991). For such substorms exhibiting multiple BBFs, it is challenging to confirm the wedgelet idea from in situ data due to sparse spacecraft coverage.

Regional magnetotail simulations (Birn & Hesse, 2013, 2014a; Birn et al., 2019) do provide support for the SCW composition by wedgelets. However, this problem has not yet been considered in the context of a self‐consistent global magnetosphere simulation unencumbered by artificial boundaries in the magnetotail and including upstream solar wind (SW) driving and coupling with the ionosphere. In this paper, we employ such a magnetohydrodynamic (MHD) global magnetosphere model with the capability to resolve mesoscale plasma sheet flows (BBFs) and elementary dipolarizations (DFs), excluding their kinetic structure (e.g., Sergeev et al., 2009). We do not address the composition of the SCW by wedgelets per se, leaving specifically the generation of field‐aligned currents by localized flows and their ionospheric closure outside of the present discussion. Instead, we use our simulation to determine quantitatively whether a global (in magnetic local time, MLT) substorm dipolarization in the inner magnetosphere (within 8 *R*_E_) is a result of an accumulation of many localized dipolarizations.

To address these questions, we perform a simulation of a real substorm event described below. This allowed us to confirm the realism of our results by comparing the properties of the simulated plasma flows and magnetic field dipolarizations with in situ measurements from four magnetospheric spacecraft, which were fortuitously aligned azimuthally at or near the geosynchronous orbit. While it would be futile to attempt to obtain a one‐to‐one correspondence because of the sporadic nature of BBFs and DFs, we are looking for a general confirmation that the magnitudes of the flow and field variations in the simulation are in accord with those measured. The results of this exercise not only give us confidence that our conclusions are applicable to the real system but also enabled us to cross‐examine some detailed properties of the simulated BBFs and DFs with those observed by the magnetospheric spacecraft.

The paper is organized as follows. In section 2 we describe the event and the upstream SW data used to drive the simulation. Sections 3 and 4 describe the simulation method and the magnetospheric data sources, respectively. Section 5 presents the results of the paper. In section 5.1 we overview the results of the simulation before delving into a more quantitative analysis of the simulated near‐Earth flows, dipolarizations, and magnetic flux transport in section 5.2. In section 5.3 we present the results of model‐data comparisons and take a more detailed look at the properties of individual dipolarizations in the simulation and data, respectively. In section 6 we discuss the results and, finally, section 7 concludes the paper.

## 2 Event Description

**Figure 1:**
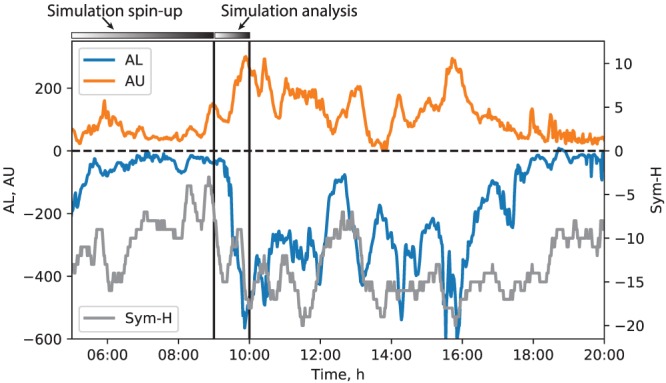
Overview of the geomagnetic indices on 9 August 2016 between 05:00 and 20:00 UT from the National Aeronautics and Space Administration OMNI database. The horizontal bars at the top, along with the vertical lines placed at 09:00 and 10:00 UT, indicate the duration of the simulation presented below (see also Figure 2). The simulation covered the 05:00–10:00 UT interval, where the 09:00–10:00 UT 1‐hr interval was used for analysis and the rest for preconditioning. The figure indicates that the 09:00–10:00 UT interval covered partly the growth phase, the entire expansion phase, and the beginning of the recovery phase of the simulated substorm. The rest of the 05:00–20:00 UT interval is shown to demonstrate the overall geomagnetic activity context on that day.

To avoid conflating storm time and non–storm time dynamics, we consider an isolated substorm that occurred after ∼08:00 universal time (UT) on 9 August 2016, studied recently observationally by Panov et al. (2019). Figure 1 shows the auroral electrojet indices (*AU* and *AL*) along with the storm time *Sym*‐*H* index. To place the simulation discussed below in the overall context of geomagnetic activity on that day, the figure covers a multihour interval that is substantially longer than the period covered by the simulation. Before the expansion phase of the substorm (starting after 09:00 UT), the *AL*/*AU* indices were insignificant for more than 3 hr. The expansion phase ended at about 09:57 UT, when the *AL* index magnitude started to decrease. According to the *AL* index, the recovery phase was interrupted by further auroral activity starting after ∼11:00 and lasting until ∼18:00 UT, after which the geomagnetic activity subsided. The *Sym*‐*H* index magnitude remained below ∼20 nT throughout the simulated period affirming that the ring current was inconsequential to magnetospheric dynamics. Accordingly, we use a standalone global MHD model of the magnetosphere without including a coupled ring current component (section 3).

**Figure 2:**
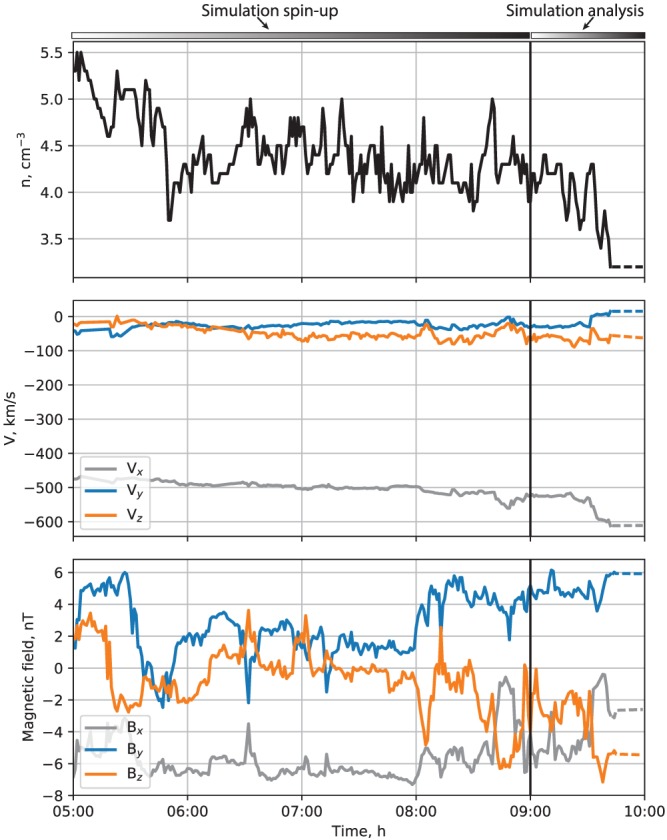
Solar wind and interplanetary magnetic field (IMF) parameters on 9 August 2016 from the National Aeronautics and Space Administration OMNI database. The vector variables are presented in the solar magnetic coordinates, in which the simulation is performed. The observed IMF *B*_*x*_ component is shown, but it was set to 0 in the simulation. Note also that no solar wind/IMF data were available at the end of the interval; the simulation was driven by the parameters fixed to the last available value during this time (in the geocentric solar ecliptic coordinates), which is indicated by the dashed line segments toward the end of the plotted interval. The horizontal bars at the top indicate the time intervals used for preconditioning (05:00–09:00 UT) and for analysis (09:00–10:00 UT). The vertical line marks the beginning of the analysis period, 09:00 UT.

Figure 2 shows the SW density and velocity, and interplanetary magnetic field (IMF), respectively. All vector components are given in the solar magnetic (SM) coordinate system, as they are input into the simulation, except the *B*_*x*_ component, which was set to 0. Prior to the substorm onset around 09:20 UT (identified from the *AU*/*AL* indices; see Figure 1), the SW remained fairly steady with the number density hovering between 4 and 5 cm^−3^ and velocity of ∼500 km/s predominantly in the earthward direction. The IMF was more variable with both most geoeffective components (*B*_*y*_ and *B*_*z*_) exhibiting fluctuations and occasional sign flips. After about 08:30 UT, the IMF *B*_*z*_ turned southward and intensified before returning to almost 0 nT around 09:00 UT and then turning southward again. It is these dynamics that presumably resulted in the observed substorm after 09:20 UT.

The results presented below concentrate on the 09:00–10:00 UT 1‐hr period including partly the growth, the entire expansion phase, and the first few minutes of the recovery phase of the substorm. It should be noted that there was a gap in the OMNI data after about 09:40 UT, during which time the simulation was driven with constant SW/IMF values as shown in Figure 2. Since changes in the upstream driving would take time to propagate to the nightside geosynchronous region and, in any event, our detailed comparisons with data focus on the times prior to 09:40 UT, this gap in the OMNI data is inconsequential to the discussion below. Moreover, our conclusions regarding the nightside dipolarization are general in nature and do not rely on specifics of the upstream driving.

## 3 Simulation Method

We used the Lyon‐Fedder‐Mobarry (LFM) model (Lyon et al., 2004; Merkin & Lyon, 2010) run at high resolution that affords grid sizes of Δ*z*≈0.1 *R*_E_ in the inner plasma sheet (around *x*∼−20 *R*_E_; Merkin, Anderson, et al., 2013; Merkin, Lyon, & Claudepierre, 2013; Wiltberger et al., 2015). The numerical algorithms underlying the LFM code were recently described in significant detail by Zhang et al. (2019). The LFM model has been demonstrated to reproduce the fundamental loading‐unloading cycle of magnetic flux in the magnetosphere in response to southward IMF turnings (Gordeev et al., 2017). This quality, together with its ability to resolve the mesoscale plasma sheet flows and dipolarizations (Wiltberger et al., 2015), makes it an advantageous tool for the present investigation.

The outer boundary of the magnetospheric LFM grid is a cylinder with the front and back boundaries located at *x*=30 *R*_E_ and *x*=−330 *R*_E_, respectively. The radius of the cylinder, that is, the maximum extent in the *y* and *z* directions, is 124 *R*_E_. The grid is a distorted spherical one with the symmetry axis being the *x* axis pointing from Earth toward Sun. The *z* axis corresponds to the SM *z* axis, and the *y* axis completes the right‐handed coordinate system and points from dawn to dusk. The total number of grid cells is 212×192×256 in the radial, polar, and azimuthal dimensions, correspondingly.

The ionospheric boundary is treated as a thin spherical shell where the field‐aligned currents generated in the magnetosphere close via Ohm's law. The full details of this approach are given by Merkin and Lyon (2010). An ionospheric conductance model is used, which combines the solar extreme ultraviolet and auroral electron precipitation contributions (Fedder et al., 1995; Wiltberger et al., 2009). The solar *F*10.7‐cm flux index was set to 100, which resulted in noon Northern hemisphere Pedersen and Hall conductances of ∼9°S and ∼13°S at 35° magnetic latitude, respectively. The nightside auroral zone conductances were highly variable in response to the changing conditions in the plasma sheet and field‐aligned currents, and the Hall conductance was generally higher than Pedersen by a factor of 2–3. Around substorm onset (∼09:23 UT), the auroral Pedersen and Hall conductances at midnight were 6°S and 16°S, respectively.

The MHD simulation was started at 05:00 UT and thus preconditioned for 4 h with the observed upstream SW/IMF parameters (Figure 2) prior to 09:00 UT when the period of interest (09:00–10:00 UT) begins. The simulation data were saved at a 5‐s time cadence to capture the temporal evolution and the time profiles of fast‐moving BBFs and the sharp structure of DFs embedded in them.

## 4 Data

For magnetospheric data, we used magnetic field from the National Oceanic and Atmospheric Administration Geostationary Operational Environmental Satellites' (GOES) Space Environment Monitor Magnetometers (https://www.ngdc.noaa.gov/stp/satellite/goes; see also GOES N SERIES DATA BOOK, Revision D, February 2010) and from the fluxgate magnetometers of the FIELDS instrument suite (Torbert et al., 2016) onboard the National Aeronautics and Space Administration MMS (Burch et al., 2016). For plasma data, observations by the Fast Plasma Instruments (Pollock et al., 2016) were used.

## 5 Results

In what follows, for brevity, we call the simulated abrupt reconfiguration of the magnetosphere a “substorm” and the start of the global dipolarization near the geosynchronous orbit the “substorm onset.” As shown in section 5.3 below, the simulated behavior of the magnetosphere near the geosynchronous orbit on the night side was similar to that observed in terms of the magnetotail stretching in the growth phase and its abrupt dipolarization in the expansion phase. These are classical signatures of a magnetospheric substorm (e.g., Kepko et al., 2015; Sitnov et al., 2019), which gives justification to the above semantic choices.

### 5.1 Simulation Overview

**Figure 3:**
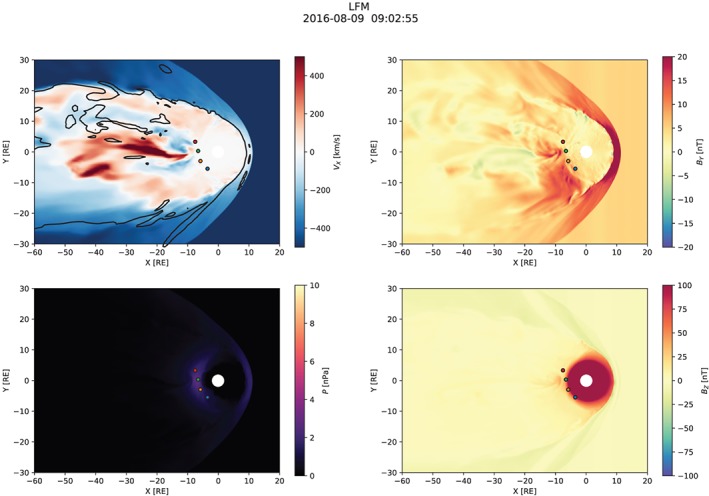
Overview of the simulation ∼20 min before the substorm onset. The solar magnetic equatorial plane is shown in each panel. Magnetospheric spacecraft are marked with blue (GOES‐13), orange (GOES‐14), green (GOES‐15), and red (MMS‐1) circles which indicate the spacecraft positions projected to the plane. The black contour in the upper left panel indicates the *B*_*z*_=0 isocontour. LFM = Lyon‐Fedder‐Mobarry.

**Figure 4:**
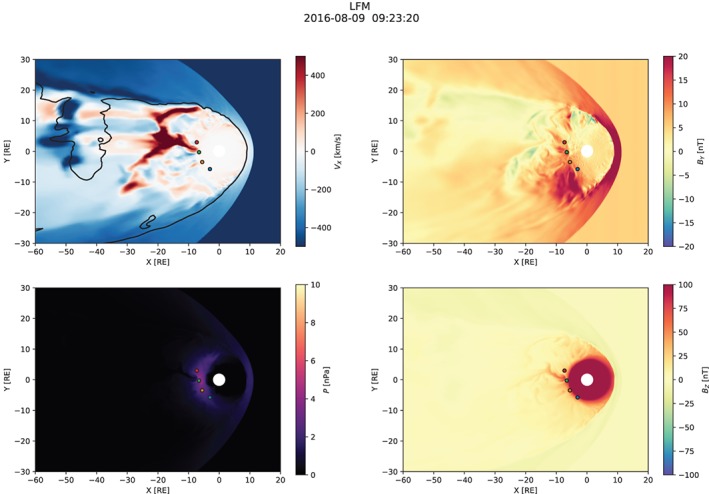
Simulation overview around the time of the substorm onset. Same format as in Figure 3.

**Figure 5:**
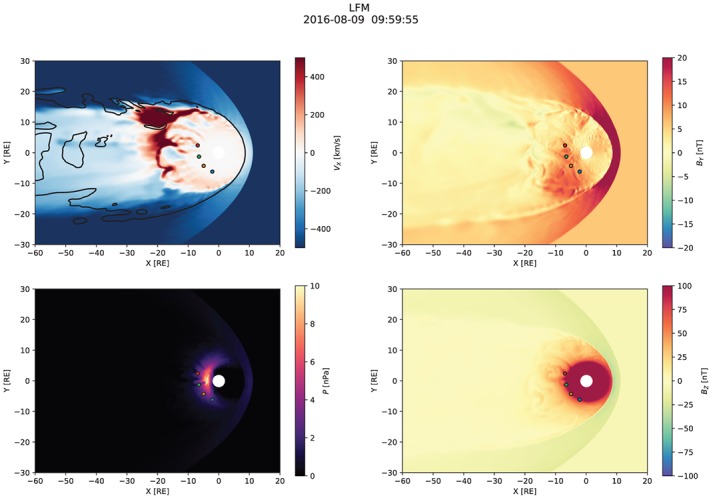
Simulation overview at the end of the simulation, in the early recovery phase ∼40 min after the substorm onset. Same format as in Figure 3.

Figures 3, 4, 5 show snapshots from the simulation overview “Movie S1” included in supporting information (SI). All figures have the same format and show the equatorial distribution of plasma and magnetic field variables. In all panels, four spacecraft are indicated: GOES‐13, GOES‐14, and GOES‐15, and MMS‐1. Comparisons with their measurements are presented below (section 5.3).

Figure 3 shows the magnetospheric configuration in the substorm growth phase, approximately 20 min prior to the onset. The divergent *V*_*x*_ flows indicate the existence of an X‐line somewhere in the midtail (beyond ∼40 *R*_E_ near midnight), but its exact location is unclear from the figure (nor essential to the subsequent discussion) because it may be located away from the SM equator due to significant IMF *B*_*y*_ and non‐*V*_*x*_ SW velocity components (note that the *B*_*z*_=0 contour in the *V*_*x*_ panel is shown to guide the eye but does not necessarily coincide with the X‐line since the simulation lacks north‐south symmetry.) This time instance is interesting because it indicates a significant BBF penetrating close to the geosynchronous orbit near midnight with its earthward edge reaching the GOES‐15 satellite. Also evident are strong rebound flows (Ohtani et al., 2009) on either side of the penetrating BBF such that MMS equatorial projection (red circle) is close to being engulfed in the tailward flow. This flow configuration is similar to that seen in regional magnetotail simulations of BBFs (Birn et al., 2011). The pressure distribution is relatively unremarkable at this time and is shown for contrast with the corresponding panels in Figures 4 and 5 indicating significant pressure buildup relative to this preonset state. The upper right panel shows that *B*_*y*_ in the magnetotail is positive as expected for positive IMF *B*_*y*_ (e.g., Petrukovich, 2011) but at the geosynchronous orbit it is quite weak at this time. The bottom right panel shows the arrival of the DF associated with the BBF mentioned above to the dipole‐dominated region indicated by the saturated red color (although the perceived location of the boundary between the dipole and tail‐like magnetic fields is obviously strongly affected by the choice of the color palette.)

Figure 4 shows the simulated magnetospheric configuration at the time near the onset. The most pronounced difference from the previous time (cf. Figure 3) is the formation of a new X‐line closer to the earth and skewed toward the duskside. The new X‐line is located somewhere tailward of *x*=−20 *R*_E_ and is indicated by the intense divergent earthward‐tailward flows as well as the large‐scale break in the *B*_*z*_=0 contour suggesting a change in the magnetic field topology. The earthward flows break up into multiple intense channels (BBFs), most pronounced near the midnight and at the dusk flank. The midnight BBF penetrates to the GOES‐15 location, and it is this BBF, along with the associated DF, that starts the global substorm dipolarization (see below). The localized dipolarization associated with the midnight BBF is evident with the characteristic “mushroom” shape discernible also in the regional magnetotail simulations by Birn et al. (2011), see upper left panel in their Figure 8, in particular). We return to this particular BBF in more detail in section 5.3. Lastly, a noticeable increase in the inner magnetosphere pressure is evident in comparison with the previous time (Figure 3) indicative of enhanced convection and more frequently penetrating BBFs.

Finally, Figure 5 shows a snapshot of the simulation in the recovery phase of the substorm. At this time, a fairly regular azimuthally global X‐line has formed in the tail around *x*=−30 *R*_E_ indicated, again, by the divergent earthward‐tailward flows. The earthward flows break up into azimuthally localized channels (BBFs) that generally penetrate to geosynchronous orbit and rebound as is evident from intermittent weak tailward flows between, roughly *R*_*xy*_=6 *R*_E_ and *R*_*xy*_=15−20 *R*_E_ on the nightside, where *R*_*xy*_ is the geocentric distance in the plane. The magnetic field has further dipolarized at the spacecraft locations and, more generally, across a broad range of MLT on the nightside. This is the result of the global substorm dipolarization discussed in detail below. The thermal pressure distribution now shows a significant enhancement relative to the preonset level (Figure 3) with the peak well within the geosynchronous orbit and skewed slightly toward the premidnight sector.

### 5.2 Quantitative Analysis of Near‐Earth Dipolarization

**Figure 6:**
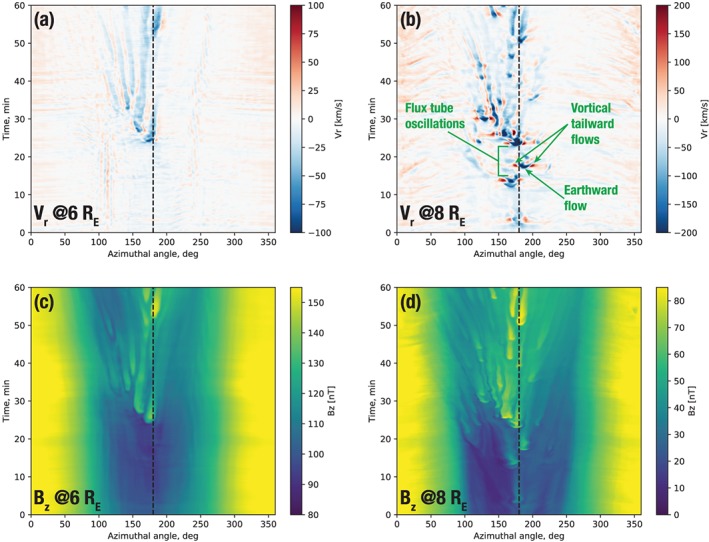
Stack plots of the simulated radial velocity (a, b) and *B*_*z*_ magnetic field component (c, d) as functions of time and azimuthal angle (*ϕ*=0°, 360° corresponds to noon; *ϕ*=180° to midnight; *ϕ*<180° to dusk sector; and *ϕ*<180° to dawn sector) at a fixed radial distance. Panels (a and c) and (b and d) correspond to 6 and 8 *R*_E_, respectively. The vertical axis indicates time in minutes elapsed since 09:00 UT on 9 August 2016. The dashed vertical line marks midnight in all plots.

In this section we address quantitatively the contribution of azimuthally localized flows (BBFs) and magnetic field dipolarizations (DFs) to the overall dipolarization of the inner magnetosphere during the expansion phase of the substorm. Figure 6 shows the simulation results in a form intended to elucidate this question. Note that, in all figures in this section, time indicates minutes elapsed since 09:00 UT.

In all panels in Figure 6 we took a slice through the simulation at a fixed radial distance (6 and 8 *R*_E_) in the equatorial plane and plotted the result as a function of time. The panels in the top row (a, b) show such a representation of the radial flow velocity, *V*_*r*_. The arrival of multiple BBFs is evident in both panels, and it is also clear that the first three major BBFs ocurring before *t*=20 min do not make it to the 6 *R*_E_ radial distance. The first of them is the one that we already observed in Figure 3. However, starting after *t*=20 min, multiple BBFs penetrate to 6 *R*_E_, the first of which is the one depicted in Figure 4 near midnight. We refer to this time as the substorm onset, which is defined more quantitatively below. Figure 6b shows also that at 8 *R*_E_ each earthward moving BBF (indicated by the blue color) is accompanied by two tailward moving flows flanking it marked by the red color. These flows are part of the vortical BBF structure (Birn et al., 2011; Panov et al., 2010). There is also evidence of an oscillating flow, where the BBF overshoots its equilibrium position and oscillates until settling in, in agreement with past theory, observations, and simulations (Chen & Wolf, 1999; Panov et al., 2010; Wolf et al., 2012). We return to this feature of the simulation below. Figures 6a and 6b suggest that whatever radial magnetic flux transport occurs at these distances, it is mediated by the azimuthally localized flow channels, since everywhere outside of these channels the radial flow velocity is essentially 0. Another noteworthy detail is the duskward bias of the BBFs, which is likely due to the skew of the near‐Earth X‐line (see Figures 4 and 5).

Figures 6c and 6d depict the vertical component of the magnetic field, *B*_*z*_, in the same format. These panels demonstrate an avalanche‐like accumulation of the magnetic field on the nightside. At 8 *R*_E_ (panel d) the magnetic field starts accumulating already with the impact of the precursor BBFs before *t*=20 min but the BBF frequency increases after this time, and as a result of these multiple dipolarizations the overall magnetic field grows substantially across the nightside between *t*=0 min and *t*=60 min (i.e., between 09:00 and 10:00 UT). Figure 6c leads to the same conclusion although, in agreement with panel (a), the DFs only start penetrating to this radial distance after t=20 min.

**Figure 7:**
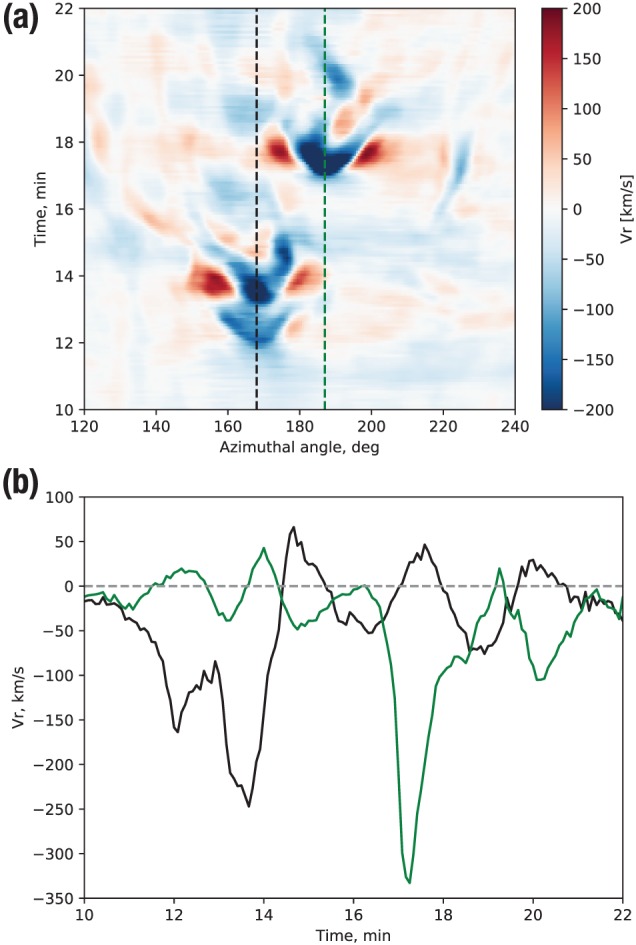
(a) Stack plot of the simulated radial velocity *V*_*r*_ as function of time and azimuthal angle at 8 *R*_E_ radial distance (zoom‐in in Figure 6b.) (b) Two vertical slices through the plot in panel (a) at the dashed lines indicated there.

To digress for a moment toward the flux tube oscillations mentioned above, we show in Figure 7 a zoom‐in on the corresponding region highlighted in Figure 6b. The figure shows a BBF arriving after *t*=12 min between 160° and 180° azimuthal angle (note the spatial substructure within the BBF resulting in the double peak of the earthward flow.) After the earthward flow subsides and the BBF stops (this can be confirmed in Movie S1 and its zoomed‐in Version S2 in the SI), the flow exhibits clear earthward‐tailward oscillations. The black dashed line in Figure 7a runs right through this oscillating region, while the green dashed line is shifted by 19° in azimuth. The corresponding *V*_*r*_(*t*) traces are shown in Figure 7b, indicating clearly the *V*_*r*_ oscillation with a period of 2–3 min in the black trace after the earthward (*V*_*r*_<0) flow stops (in remarkable resemblance to THEMIS observations by Panov et al., 2010, Figure 2d). The green trace is shown for comparison to indicate what would a virtual spacecraft just ∼2.65 *R*_E_ to the side see in this case. Clearly, the primary feature that it observes is the other BBF arriving at *t*≈17 min around 187° longitude, but no clear signature of flux tube oscillations that are evident in the black trace. Figure 7 thus demonstrates first how fortuitous should a spacecraft location be to observe such an oscillating structure (capturing both the radial distance of the flux tube equilibrium location and its localization in azimuth) and, second, that the azimuthal size of the BBF is in agreement with prior observational estimates (Angelopoulos et al., 1996; Sergeev et al., 1996; Nakamura et al., 2004; Liu et al., 2015).

**Figure 8:**
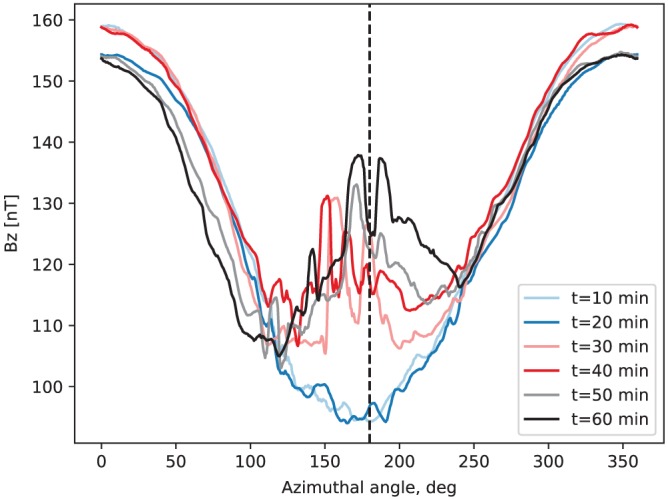
Dependence of the simulated *B*_*z*_ magnetic field component on the azimuthal angle at 6 *R*_E_ for the times indicated in the legend. These traces are equivalent to slicing Figure 6c horizontally at the corresponding times. The vertical dashed line marks midnight.

Returning to the discussion of the near‐Earth dipolarization, Figure 8 depicts the *B*_*z*_ dependence on the azimuthal angle for six different times at 6 *R*_E_. The abrupt dipolarization of the field is now obvious between *t*=20 min (blue trace) and *t*=30 min (light‐red trace). Furthermore, while midnight and premidnight exhibit a rather complex magnetic field evolution after the onset due to the constant bombardment by the BBFs and DFs (cf. Figure 6), on the morning side a slower (relative to the abrupt onset) *B*_*z*_ increase is evident due to the azimuthal convection of the magnetic flux brought from the tail near midnight.

**Figure 9:**
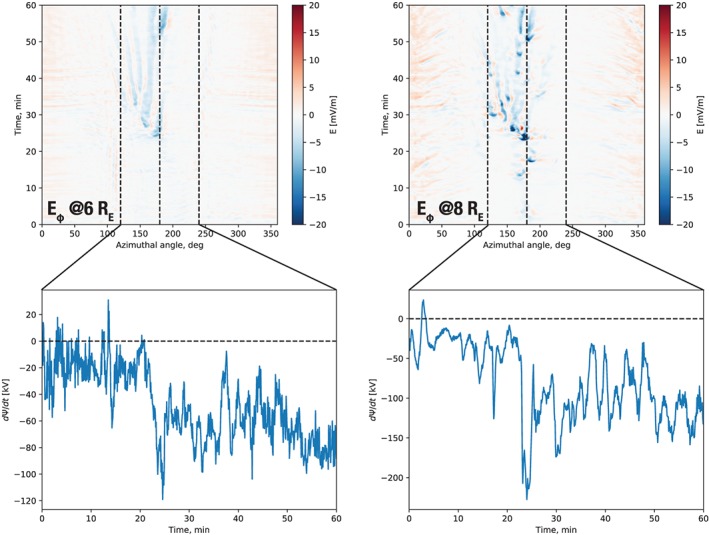
The top panels are similar to Figure 6 except the azimuthal component of the electric field, *E*_*ϕ*_, is shown at 6 and 8 *R*_E_ circles. The figures at the bottom integrate *E*_*ϕ*_ between 20:00 and 04:00 magnetic local time (indicated by dashed lines in the top plots along with midnight) to obtain the time dependence of the magnetic flux Ψ transport rate.

To address specifically the question of magnetic flux transport from the tail in a more quantitative fashion, we plot in Figure 9 the azimuthal electric field, *E*_*ϕ*_=*V*_*r*_ *B*_*z*_, in the same format as Figure 6. The top panels in this figure confirm that indeed all magnetic flux transport at both 6 and 8 *R*_E_ occurs within the azimuthally localized flow channels (BBFs), since virtually no convective electric field exists outside of them. In the bottom panels of Figure 9 we integrate *E*_*ϕ*_ between 20:00 and 04:00 MLT to assess the total magnetic flux transport rate $d\Psi /dt=R_{xy}\int_{20}^{04}E_{\phi }d\phi$ toward Earth, where *R*_*xy*_=6 or 8 *R*_E_ is the radial distance in the equatorial plane (negative dΨ/d*t* corresponds to earthward transport under this definition). A few points are evident from these plots. First, the onset is clearly discernible just after *t*=20 min as an abrupt increase in the flux transport rate at both radial distances. Second, the integrated magnetic flux transport is all earthward, despite the existence of tailward rebound flows. Third, not only does the earthward flux transport occur exclusively via the azimuthally localized flow channels, it is also bursty in time even after integrated over azimuth.

**Figure 10:**
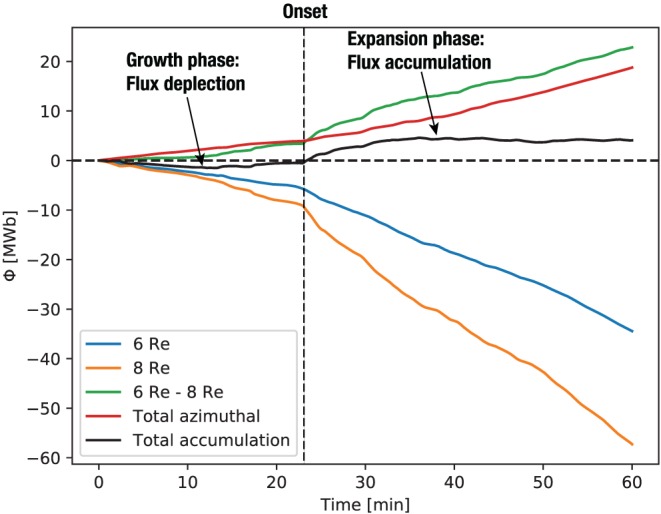
Integrated magnetic flux transported through the boundaries of the wedge region in the equatorial plane bounded by 20:00 and 04:00 magnetic local time on the sides and 6 and 8 *R*_E_ along the radial direction. The black trace indicates the sum of all fluxes, that is, the total amount of magnetic flux accumulated in the wedge region over the 1 h period of the simulation (09:00–10:00 UT).

Finally, Figure 10 depicts the total flux transport balance through the equatorial wedge region bounded by the 6 and 8 *R*_E_ circles and the 20:00 and 04:00 MLT radial lines. The orange trace shows the cumulative earthward (negative) magnetic flux transport through the 8‐*R*_E_ arc, while the blue trace shows the same for the 6‐*R*_E_ arc. Clearly, much more flux flows earthward through the 8‐*R*_E_ boundary than through the 6‐*R*_E_ boundary. The green trace shows the imbalance between the two. To check how much of this unbalanced flux accumulates in the wedge region versus escaping azimuthally, we also calculate the azimuthal flux transport through the side (fixed MLT) boundaries of the wedge. The result is the red trace (positive here means azimuthal transport out of the wedge region), which demonstrates an interesting detail: Before the onset, the azimuthal outflow from the wedge region exceeds the total radial inflow leading to the overall flux depletion; on the contrary, after the onset, the total radial influx surpasses the azimuthal outflow leading to the accumulation of the magnetic flux in the wedge region. This is indicated by the black trace, which is the difference between the green and red traces. Once presented this way, the simulation data clearly delineate the substorm onset as the time where the cumulative flux (black trace) changes sign. These results are consistent with the previous regional magnetotail simulations by Hsieh and Otto (2015) and global simulations using the LFM code (Gordeev et al., 2017).

### 5.3 Comparison With Observations and Detailed Structure of Near‐Earth Dipolarizations

**Figure 11:**
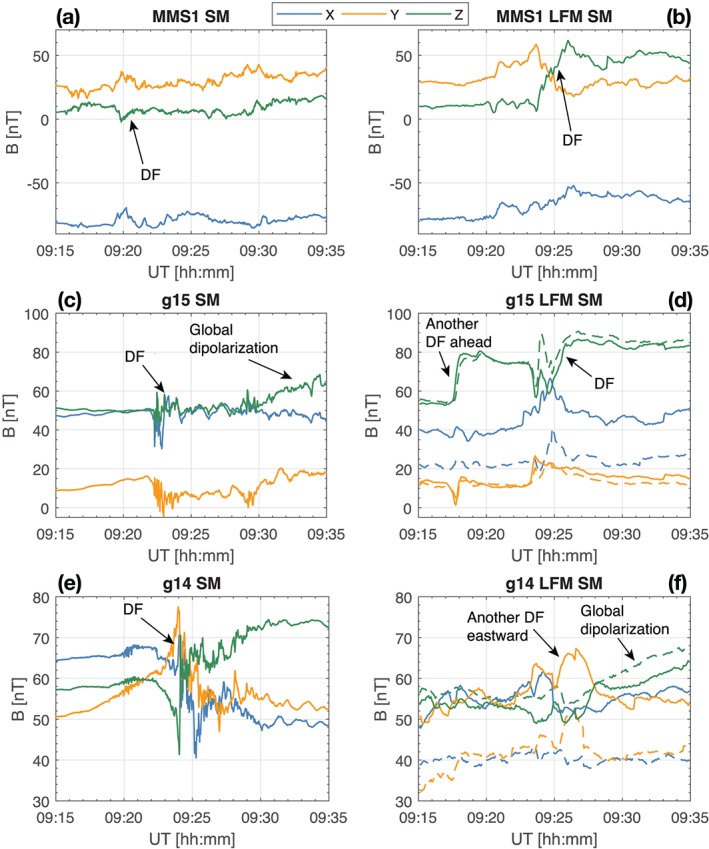
(left column) MMS‐1 (a), GOES‐15 (c), and GOES‐14 (e) SM magnetic field components on 9 August 2016 for the time period indicated on the horizontal axis. (right column) The same magnetic field components obtained from the simulation. The virtual MMS‐1 data are plotted along the actual spacecraft trajectory as are the GOES‐14 and GOES‐15 data indicated by the dashed lines in panels (d) and (f). The solid lines in the GOES‐14 and GOES‐15 panels are plotted along the virtual spacecraft trajectory raised by Δ*z*=0.4 *R*_E_ in the SM *z* direction. DF = dipolarization front; LFM = Lyon‐Fedder‐Mobarry; SM = solar magnetic.

Figure 11 shows a comparison between the observed and simulated magnetic field components at the three spacecraft that are closer to midnight (GOES‐14, GOES‐15, and MMS‐1; see Figures 3, 4, 5for their equatorial positions). Figure 11 concentrates on 20 min around the onset, while Figure S1 complements it by showing the same comparisons for the full 09:00–10:00 UT period, with the addition of the GOES‐13 satellite located further dawnward. We first note that both the simulation and observations exhibit the magnetic field dipolarization corresponding to the substorm onset at approximately the same time, after 09:20 UT (cf. auroral indices in Figure 1). In the model, the dipolarization is due to the arrival of a BBF from a newly formed X‐line, which might indicate that a similar process occurred in nature. Figures 11 and S1 further demonstrate that most substorm activity, both observed and simulated, occurred in the MLT range between MMS‐1 and GOES‐14.

While we discuss the time around the onset in more detail below, we remark here on two primary details seen in Figure S1. First, in the growth phase (before 09:20 UT), both in the simulation and observations all spacecraft indicate a stretching of the magnetic field (decreasing *B*_*z*_). Note also that since MMS was in the southern lobe, the field stretching is less pronounced at its location than at the more equatorial GOES satellites. The stretching is also accompanied by the intensification of *B*_*x*_ (*B*_*y*_ at GOES‐13 due its dawnward location) consistent with magnetic flux loading into the lobes. The simulation tends to underestimate *B*_*x*_ (and *B*_*y*_ at GOES‐13 and GOES‐14), which we discuss below. Second, toward the end of the simulation (10:00 UT) both the data and simulation indicate a significant increase in *B*_*z*_ at all spacecraft locations, indicating a global dipolarization. The magnitude of the dipolarization, and the degree of agreement between the simulation and observations, varies between the spacecraft, but what is important is that the overall strength of dipolarization is consistent between the model and data, which was the primary purpose of this comparison and gives credence to the simulation results reported above.

**Figure 12:**
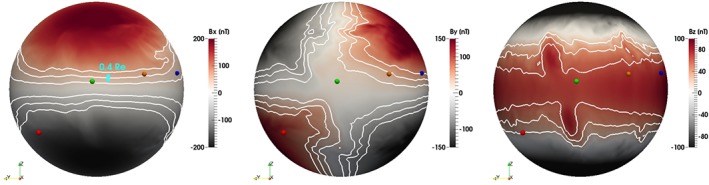
Magnetic field *B*_*x*_ component at 09:15 UT color coded on a spherical slice through the simulation at the radial distance of 6.4 *R*_E_. The view is from a vantage point on the solar magnetic *x* axis down the tail looking toward Earth; that is, dusk is to the left and dawn is to the right. The spacecraft are shown with small spherical glyphs color coded the same way as in Figures 3, 4, 5: MMS‐1 (red), GOES‐15 (green), GOES‐14 (orange), and GOES‐13 (blue). The white contours indicate |*B*_*x*_|=20, 40, and 60 nT, in the order of increasing distance from the magnetic equator. The vertical cyan bar indicates the 0.4‐*R*_E_ scale, that is, the shift used for GOES‐14 and GOES‐15 satellites in Figure 11 (see text for details).

We now turn to a more detailed discussion of Figure 11. The top row of panels shows the comparison between the observed and simulated data at the MMS‐1 spacecraft. Prior to the onset all three of the magnetic field components agree exceptionally well, indicating that the amount of magnetic flux loaded into the lobes during the growth phase in the simulation was consistent with reality. At GOES‐15 prior to onset both *B*_*y*_ and *B*_*z*_ also agree quite well with those observed but *B*_*x*_ is underestimated at the actual spacecraft location (dashed lines). For GOES‐14, which is more dawnward, *B*_*y*_ is also underestimated. Since in this region the dipole magnetic field is still strong but quickly changing, a small shift in the spacecraft location will make a large difference in the observed field. To take this possibility into account, we shifted the virtual GOES‐14 and GOES‐15 spacecraft by 0.4 *R*_E_ in the positive SM *z* direction. The data from thus modified virtual spacecraft are plotted with solid lines in panels (d) and (f) and show a much better agreement with the observations. Such a displacement of the virtual spacecraft could occur if the nightside magnetic configuration was shifted slightly southward (e.g., due to the presence of a negative SW *V*_*z*_; cf. Figure 2) or if the field was somewhat more stretched in reality than in the simulation. Figure 12 demonstrates the magnetic field configuration in the simulation along with the spacecraft locations indicating, in particular, the MMS‐1 position in the southern lobe (note that it is located ∼1.7 *R*_E_ above the shown spherical slice, while the GOES satellites are very close to it). The figure complements Figures 3, 4, 5 by depicting the *z* coordinates of the spacecraft and confirms that the coordinate shift required for better agreement with the measurements in Figure 11 is quite small relative to the local scale size of the magnetic field gradient. For the interested reader, Figure S2 in the supporting information shows the *B*_*y*_ and *B*_*z*_ magnetic field components in the same format.

**Figure 13:**
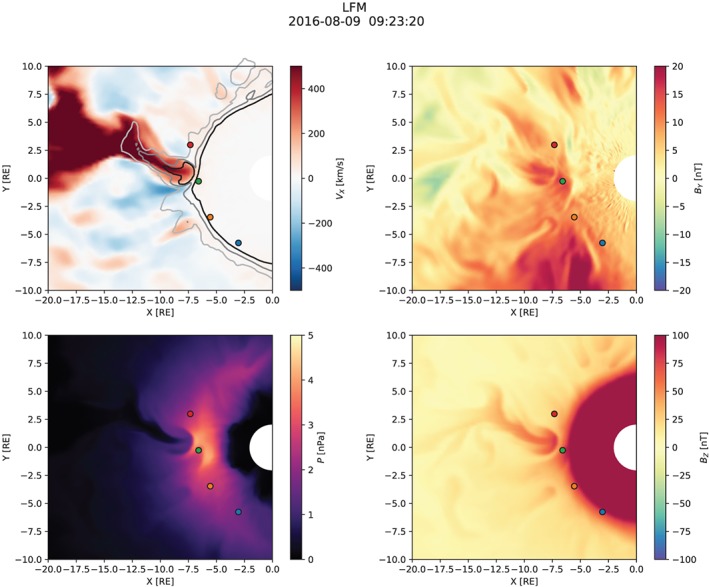
A version of Figure 4 zoomed‐in on the protruding bursty bulk flow initiating the substorm in the simulation. The upper left panel additionally shows the *B*_*z*_=25‐, 40‐, and 55‐nT isocontours (light gray, medium gray, and black, correspondingly). LFM = Lyon‐Fedder‐Mobarry.

Returning to Figure 11, the virtual MMS‐1 spacecraft observed a clear DF marked in panel (b). Figure 13 shows a zoomed‐in view of Figure 4 (see also Movie S2). In addition to Figure 4, Figure 13 also shows the *B*_*z*_=25, 40, and 55 nT (light gray, medium gray, and black, correspondingly) isocontours in the equatorial plane. The figure along with Movie S2 indicates that the equatorial projection of the MMS probe (the red spherical glyph) was disturbed by the tailward return flow and a concomitant magnetic field fluctuation (more on this below.) However, since the probe was located significantly southward of the magnetic equator, it actually observed a dipolarization corresponding to the increased *B*_*z*_ in the tailward portion of the DF seen in Figure 13 hitting the location of the GOES‐15 probe around the same time (also marked in panel d). Note that this tailward side of the dipolarization develops into what could be construed as another, broader BBF/DF by 09:25 UT (see Movie S2; see also Figure 15d below.) The magnetic variations observed in reality by GOES‐15 and MMS‐1 (marked with “DF” in Figures 11a and 11c) had a smaller amplitude than in the simulation, and, based on the energetic electron data at the geosynchronous spacecraft, Panov et al. (2019) interpreted them as parts of the same DF observed slightly later by GOES‐14 (Figure 11e). In the simulation, however, GOES‐14 did not observe the same DF as GOES‐15 and MMS‐1 but rather was impacted by another, smaller BBF/DF around 09:25 UT (see Movies S1 and S2) with most pronounced increase in the *B*_*y*_ component (Figure 11f).

**Figure 14:**
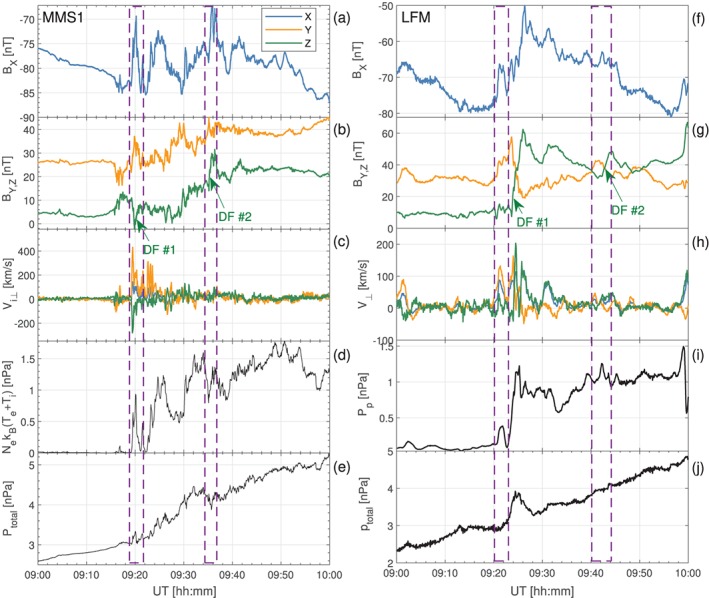
(left column) MMS‐1 data on 9 August 2016 between 09:00 and 10:00 UT. Shown at survey cadence are as follows: B_*x*_ (a) and B_*y*_, B_*z*_ (b) solar magnetic magnetic field components; three solar magnetic components of the perpendicular ion velocity with positive *V*_⊥,*x*_ values towards Earth (c); sum of the electron and ion thermal pressures (d); and sum of the magnetic pressure and the thermal particle pressure (e). (right column) The corresponding virtual MMS‐1 data sampled along the spacecraft trajectory through the simulation (f–j). DF = dipolarization front; LFM = Lyon‐Fedder‐Mobarry.

To summarize the above, in reality GOES‐14 observed a clear strong dipolarization similar to “Type II” dipolarizations or explosive growth phase discussed by Ohtani et al. (1992), while MMS‐1 and GOES‐15 might have been impacted by it peripherally. In the simulation, it was GOES‐15 that observed the most direct impact by the BBF initiating the substorm, while MMS‐1, located southward of the magnetic equator, observed the tailward part of this dipolarization and GOES‐14 was not impacted by it at all. Note also that the virtual GOES‐15 spacecraft saw another direct DF impact at around 09:18 UT.

**Figure 15:**
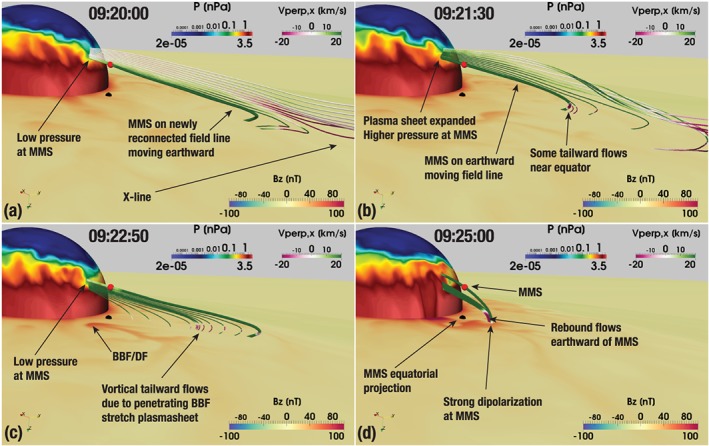
A 3‐D view of the simulated magnetosphere at the beginning (a), in the middle (b), and at the end (c) of the time period marked with the first magenta rectangle in Figure 14. (d) The same view at the later time when MMS‐1 observes the strong dipolarization. The view is from a vantage point southward of the magnetic equator in the morning sector. The axis widget in the lower left corner helps to orient the reader; in particular, it indicates the solar magnetic *z* axis pointing down. The MMS‐1 location is marked with the red spherical glyph as in the previous figures. Its equatorial projection is marked with the black spherical glyph. The equatorial plane is color coded with the solar magnetic *B*_*z*_ magnetic field component and the spherical slice through the simulation at 6.4 *R*_E_ radial distance with the plasma pressure. All panels show a thick magnetic field line anchored at the MMS‐1 location. Panels (a) and (b) trace additional field lines (with reduced thickness) from a line of seed points placed southward of MMS‐1. Similarly, panels (c) and (d) trace additional magnetic field lines (also with reduced thickness) from seed points equatorward of MMS‐1. All field lines in all panels are color coded with *V*_⊥,*x*_. The corresponding color bars are shown in all panels. BBF = bursty bulk flow; DF = dipolarization front; MMS = Magnetospheric Multiscale.

The differences between the simulation and the observations are not surprising; as noted above, given the sporadic nature of BBFs/DFs, it would be unrealistic to expect a perfect agreement. However, an interesting detail to note in the data‐model comparison in Figure 11 is that qualitatively the data from the real GOES‐15 satellite correspond better with the virtual GOES‐14 satellite, and vice versa. The magnetic field signatures at the virtual GOES‐14 and GOES‐15 locations show two distinct responses. GOES‐15 observed a sharp dipolarization (in fact, two of them) corresponding to a direct hit by a BBF/DF, as discussed above. However, at the virtual GOES‐14 we observe a much slower rise in the *B*_*z*_ magnetic field component (marked as “Global dipolarization” in Figure 11f). An examination of Movies S1 and S2 reveals that this slow rise is due to a global tailward expansion of the dipolarized field region (Baumjohann et al., 1990). This similarity of the observed and simulated signatures (Figures 11c, 11f and 11e, 11d, correspondingly) leads us to conclude that the real spacecraft observed the same two types of dipolarizations, that is, a direct BBF/DF impact (GOES‐14) and a tailward expansion of the dipolarized field region (GOES‐15).

Since MMS afforded not only magnetic field but also plasma measurements, we were able to cross‐examine the plasma moments between the observations and the simulation at the spacecraft location (Figure 14). We concentrate here on a particular feature that exhibited significant similarity between the simulation and observations. The first dashed magenta rectangle, surrounding the structure denoted by the green text arrow “DF #1” in the left column in Figure 14, indicates the field and plasma disturbances associated with the DF in Figure 11a. During this time, |*B*_*x*_| exhibited a clear minimum about 15 nT deep (Figure 14a), which corresponded to a maximum of a similar amplitude in *B*_*y*_ (Figure 14b) and a maximum in the plasma pressure (Figure 14d). The positive perpendicular *x* and *y* components of the ion velocity (Figure 14c) during the |*B*_*x*_| minimum indicate earthward and duskward motion of the structure.

A similar dashed magenta rectangle in the right column in Figure 14 indicates the field and plasma disturbances just prior to the simulated DF discussed above that passed by the virtual MMS‐1 probe after ∼09:22 UT. In accordance with the MMS‐1 observations from the left column in Figure 14a, the virtual probe revealed a |*B*_*x*_| minimum about 12 nT deep (Figure 14f), which was associated with maxima in *B*_*y*_ (Figure 14g) and in the plasma pressure (Figure 14i). The simulated total pressure (Figure 14j) grew at a similar rate as observed (Figure 14e) throughout the 1‐hr interval and reached about the same level (5 nPa) by 10:00 UT. The positive perpendicular *x* and *y* components of the ion velocity during the |*B*_*x*_| minimum in Figure 14h (left magenta rectangle) manifest earthward and duskward convection of the simulated structure, consistent with the MMS observations in Figure 14c. Note that the observed and simulated DFs denoted as “DF #1” were followed by other DFs (denoted by the green text arrows “DF #2” in Figure 14) with similar magnetic and pressure structures associated with them.

Due to the clear similarity between the observed and simulated structures marked by the first magenta rectangles, and due to their apparent relationship with the corresponding DFs, we examined the simulation around this time in detail to reveal their cause. Figure 15 depicts four moments of time, picked from the simulation to correspond to the beginning (Figure 15a), middle (Figure 15b), and the end (Figure 15c) of the time period marked with the first magenta rectangle in the right panel of Figure 14. Additionally, Figure 15d shows the time a couple of minutes later when the strong dipolarization arrives at the virtual MMS‐1 spacecraft (see Figures 11 and 14). From Figure 14 it is clear that both in reality and in the simulation, MMS‐1 resided in the southern lobe prior to ∼09:20:00 UT, as evidenced by the negligible plasma pressure. Figure 15a reveals that at 09:20:00 UT the virtual spacecraft started transitioning from the lobe to the plasma sheet, which is clear from the pressure distribution on the spherical slice, but the pressure was still low. The magnetic field lines traced from seed points southward of the virtual spacecraft indicate that the field line on which MMS‐1 is located has just reconnected. Indeed, this interpretation agrees with the formation of the near‐Earth X‐line discussed above and evident in Movie S1. Due to this newly started reconnection, the magnetic flux is moving across the boundary between the lobes and the plasma sheet (note that *V*_⊥,*x*_ is earthward everywhere on the thick magnetic field line anchored at MMS‐1), resulting in the plasma sheet expansion (Ohtani & Mukai, 2006; Panov et al., 2010). This expansion causes the virtual spacecraft to transition to the plasma sheet. Figure 15b shows the moment of time when the plasma pressure at MMS‐1 is at its peak (Figure 14). At this time, the MMS‐anchored field line is moving earthward, although some tailward flows exist near the equator. The next question is what causes the pressure to drop and the magnetic field *B*_*x*_ component to increase in magnitude again just prior to the arrival of the large dipolarization at MMS‐1. Figure 15c provides the answer: At this time, the substorm‐initiating BBF/DF discussed above has already almost reached the geosynchronous orbit and the region westward of it, where MMS resides, is immersed in tailward moving flows that are part of the BBF vortical structure (see discussion above, Movies S1 and S2, and Figure 13). This tailward motion results in magnetic field stretching and plasma sheet thinning (Panov et al., 2010), which makes MMS‐1 go back to the lobe. This does not last long, however, because immediately after this the large dipolarization arrives at MMS and the plasma sheet expands again (Figure 15d).

## 6 Summary and Discussion

We demonstrated above with the help of a global magnetosphere MHD simulation that in the expansion phase of the isolated substorm considered, all plasma convection and, therefore, magnetic flux transport within 8 *R*_E_ radial distance occurred by means of azimuthally localized flow channels (BBFs) carrying significant magnetic field enhancements (DFs) penetrating to these near‐Earth distances from the plasma sheet. Figures 6 and 9 indicate the existence of rare BBFs prior to the substorm onset; however, the onset was characterized by the arrival of the most intense BBF in terms of the magnetic flux that it carried (Figure 9, bottom row after *t*=20 min). Following it were many more such localized flows that cumulatively were responsible for the global dipolarization of the inner magnetosphere about 40 min after the onset (Figure 8). This prevalence of BBFs during the growth phase is consistent with statistical observations in the magnetotail (Juusola et al., 2011). Furthermore, the BBFs/DFs occurring after the onset had distinctly different properties from those before the onset: While the latter were visible at 8 *R*_E_, only those after the onset were visible both at 8 and at 6 *R*_E_. Interestingly, the onset in the simulation was characterized by the change in the sign of the cumulative magnetic flux in a wedge region on the nightside between 6 and 8 *R*_E_ and 20:00 and 04:00 MLT. Before the onset, the total flux in this region was depleted relative to the early growth phase (09:00 UT), while after the onset the flux was elevated.

The deep penetration of BBFs in the expansion phase can probably be related to their origin in the newly formed near‐Earth X‐line responsible for the generation of flux tubes with lower entropy (Figure 4 and Movies S1 and S2 in the SI). This would be consistent with their deeper earthward penetration (Dubyagin et al., 2011). Movies S1 and S2 show the consistent growth of the thermal plasma pressure well within the geosynchronous orbit during the expansion phase due to the BBF penetration. The mechanisms of plasma heating in this ideal MHD simulation must obviously be adiabatic, but recent test‐particle simulations in similarly highly resolved MHD fields also demonstrated the importance of ion trapping and nonadiabatic effects in the transport and energization process (Ukhorskiy et al., 2018). It will be a natural continuation of this study to perform similar test‐particle simulations in the MHD fields taken from the simulation presented above. Possible non‐MHD plasma heating effects notwithstanding, the comparison with MMS plasma moments (Figures 14d and 14i) showed that the pressure in the simulation was very similar to that observed, both in terms of its magnitude and the temporal evolution during the growth and expansion phases. Another important detail about the pressure distribution in the model is that its peak was shifted to the premidnight sector. Since there is no drift or Hall physics included in our MHD simulation (cf. Lin et al., 2014; Yang et al., 2011), the location of the pressure peak must be explained by the asymmetry of the nightside flows and the premidnight skew of the near‐Earth X‐line. This, in turn, is probably a combined effect of the asymmetric upstream driving (e.g., IMF *B*_*y*_ and non‐*V*_*x*_ SW velocity components) and ionospheric conductance (Lotko et al., 2014). The asymmetry of the pressure distribution is another interesting effect to be examined with the test‐particle approach.

The amount of magnetic flux accumulated on the nightside (Figure 10) in the simulation may, at the first glance, appear small compared to prior estimates of the amount of flux transported by BBFs in the tail (e.g., Liu et al., 2014). However, we note that it is only the flux that *remains* in the wedge region between 6 and 8 *R*_E_ and 20:00 and 04:00 MLT that is small (a few megawebers). The total amount of earthward flux entering the 8 *R*_E_ region between 20:00 and 04:00 MLT (∼50 MWb over roughly 30 min after onset) is much more in line with expectations from observations in the plasma sheet (e.g., Liu et al., 2014, estimate that 135 MWb may be transported in the tail over 1‐hr time period). Considering that much of the nightside convection is actually diverted around the inner magnetosphere (cf. Figure 4), that is, ∼50 MWb is only the amount of flux that makes it to 8 *R*_E_ (not the total cross‐tail flux transport), the agreement with the above observational estimates is not surprising. In fact, the simulated electric field and flux transport rates in Figure 9 are quite similar to those observed as is the amount of magnetic field dipolarization at geosynchronous orbit (e.g., Ohtani et al., 2006).

To further probe the validity of our simulation results, we took advantage of the fortuitous alignment of three GOES satellites and the MMS spaceraft around the nightside magnetosphere (e.g., Figure 13) and performed a comparative analysis of the simulation and observations. The comparisons confirmed that the overall strength of the global dipolarization at the geosynchronous orbit in regions outside of direct DF impact (Figures 11c and 11f) was very similar. The virtual spacecraft that was more directly impacted (Figure 11d) observed a stronger dipolarization (from ∼55 to 85 nT in SM *B*_*z*_) than its counterpart in reality (from ∼58 to 72 nT; Figure 11e), but it could be either due to an overestimate of the dipolarization in the simulation or due to an impact that was not as direct in the observation. Even assuming that the simulation overestimated some individual dipolarizations, the BBF speed was underestimated, at least at the MMS location at the plasma sheet‐lobe boundary (Figures 14c and 14h), such that the electric field was more similar between the observations and the simulation. All of this gives us confidence in the conclusion that BBF/DF structures provide the dominant magnetic flux transport mechanism in the inner magnetosphere, at least during isolated substorms. Cramer et al. (2017) arrived at a similar conclusion in their simulations of geomagnetic storm events.

Our data‐model comparisons further revealed a number of important details of the dipolarization process in the inner magnetosphere. In particular, we demonstrated that there may be two types of dipolarization. One is characterized by a sharp increase in *B*_*z*_ due to a direct impact by a DF (e.g., Runov et al., 2009; Figures 11c and 11f). The other exhibits a much more gradual *B*_*z*_ increase over O(10) min (Figures 11c, 11f, and S1). This process manifests the tailward retreat of the dipole‐dominated region due to the arrival of additional magnetic flux brought by DFs elsewhere in MLT (e.g., Baumjohann et al., 1990).

**Figure 16:**
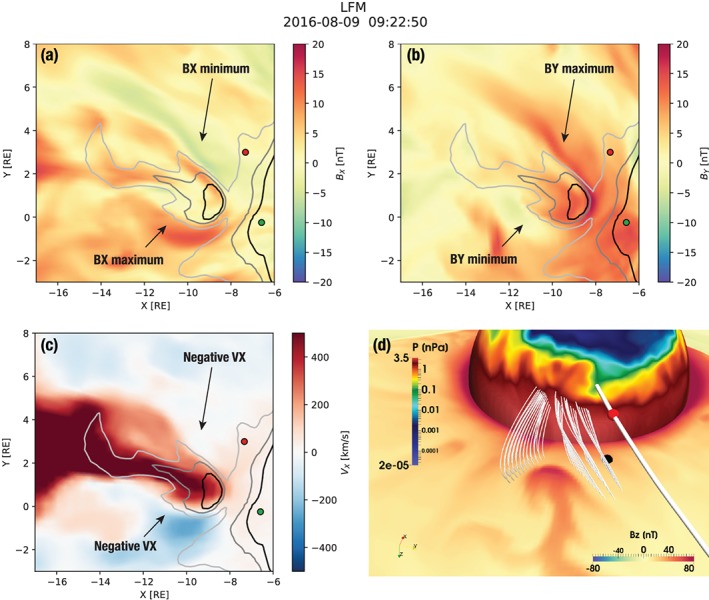
(a–c) A zoom‐in on the equatorial region of the simulation focusing on the bursty bulk flow responsible for the substorm onset. The snapshot is taken 30 s before the time in Figure 13 to emphasize different structure. The time instance is the same as in Figure 15c. Solar magnetic (SM) *B*_*x*_ (a), SM *B*_*y*_ (b), and SM *V*_*x*_ (c) are shown in the equatorial plane. Panels (a)–(c) additionally indicate the same SM *B*_*z*_ isocontours as in Figure 13: *B*_*z*_=25, 40, and 55 nT (light gray, medium gray, and black, correspondingly.) The green and red glyphs depict GOES‐15 and MMS‐1 spacecraft, respectively. (d) A 3‐D view of the simulated magnetosphere similar to Figure 15 but the view is from a vantage point southward of the magnetic equator around midnight. The axis widget in the lower left corner helps to orient the reader; in particular, it indicates the SM *z* axis pointing down. The MMS‐1 location is marked with the red spherical glyph as in the previous figures. Its equatorial projection is marked with the black spherical glyph. The equatorial plane is color coded with the SM *B*_*z*_ magnetic field component, and the spherical slice through the simulation at 6.4‐*R*_E_ radial distance with the plasma pressure. The corresponding color bars are indicated in the figure. The thick magnetic field line is anchored at the MMS‐1 location. Thinner field lines are traced from the vicinity of the head of the bursty bulk flow/dipolarization front to demonstrate their deflection.

Furthermore, we demonstrated that a spacecraft located at the high‐latitude plasma sheet boundary may observe an interesting sequence of events preceding the arrival of the dipolarization (Figure 14) that may be indicative of a complex mixture of magnetotail processes (Figure 16). First, the spacecraft transitions from the lobe into the plasma sheet due to the initiation of magnetic reconnection further in the tail followed by the plasma sheet expansion (Ohtani & Mukai, 2006). Second, if the spacecraft is located not directly in the path of the BBF but on its flank, it may transition back to the lobe because of plasma sheet stretching due to vortical tailward plasma flows associated with the BBF. Third, the spacecraft finally observes dipolarization and plasma sheet expansion due to either of the two types of dipolarization discussed above. Clearly, the above sequence of events is specific both to latitudinal and longitudinal position of the spacecraft relative to the plasma sheet boundary layer and to the intruding BBF. The example serves as a demonstration that the signal seen at a given spacecraft location may be an amalgamation of a variety of processes occurring at times simultaneously at different distances from the spacecraft.

Another example of such a process is given in Figure 16. Here we show the same moment in time as in Figure 15c. Panels (a)–(c) indicate the odd parity of the *B*_*x*_ and *B*_*y*_ components of the magnetic field on the flanks of the BBF, in regions occupied by the vortical tailward flows. Figure 16d shows the corresponding magnetic field configuration in three dimensions. The figure composition is similar to that of Figure 15, but the view is from a somewhat different vantage point, closer to midnight. Here we traced a number of magnetic field lines from the flank regions indicated in panels (a)–(c), closer to the front side of the BBF. The figure demonstrates that the magnetic field, and thus plasma, is disturbed and deflected azimuthally in front of the BBF, in accordance with the *B*_*y*_ perturbations in Figure 16b. These deflections are certain to generate field‐aligned currents and are in fact similar to magnetic field distortions reported in the context of the SCW generation in regional magnetotail simulations by Birn et al. (1999). Interestingly, in this case, the magnetic field lines are deflected not only in the azimuthal direction but also in the vertical direction, such that the cusps of the field lines are above (southward of) the equatorial plane on the left (east in this view from the southward vantage point) and below (northward of) the equatorial plane on the right (west) side of the protruding BBF. This odd parity *B*_*x*_ structure is evident in Figure 16a and is similar to plasma sheet flapping motions (Sitnov et al., 2014), although in this case it is not a signature of a plasma instability. This demonstrates that BBFs/DFs cut through the surrounding medium like an icebreaker. As a result, even spacecraft that are not located inside the flow channels see disturbances from the approaching new fronts at a distance that can significantly exceed the azimuthal size of the flow channel itself. Figure 16d shows that the MMS equatorial projection is clearly affected by this disturbance. Thus, by pushing the flux tubes equatorward of MMS toward west, in addition to their stretching due to the tailward vortical flow on the BBF flank, this process contributes to plasma sheet thinning at the MMS location and makes the spacecraft go briefly back into the plasma sheet boundary layer, as seen Figure 16d and discussed above.

## 7 Conclusions

In this paper, we carried out a high‐resolution global MHD simulation of the magnetosphere during an isolated substorm event. Through simulation analysis and cross examination with the observations by MMS and GOES spacecraft, which were fortuitously aligned in the inner magnetosphere, we have drawn the conclusions that are summarized as follows:

1. Sporadic azimuthally localized flow channels (BBFs) and magnetic field dipolarizations (DFs) were observed in the simulation during the growth phase of the substorm but did not penetrate to the geosynchronous orbit.
2. The substorm onset was characterized by an abrupt increase in the number and intensity of BBFs/DFs, which penetrated well within the geosynchronous orbit.
3. The global dipolarization of the inner magnetosphere toward the end of the substorm expansion phase was due exclusively to these azimuthally localized flows and dipolarizations. Negligible plasma convection or magnetic field transport occurred outside of these structures.
4. Over the course of the expansion phase the penetration of BBFs within the geosynchronous orbit lead to a gradual accumulation of thermal plasma pressure there. Comparison with MMS plasma moments (MMS was located away from the equatorial plane and thus effectively sampled a more distant region of the tail) showed a very good agreement between the model and observations both in terms of the magnitude and temporal evolution.
5. While the growth phase of the substorm was characterized by magnetic flux depletion in the nightside near‐Earth region (represented by a wedge‐shaped region between 6 and 8 *R*_E_ and 20:00 and 04:00 MLT in our analysis), the expansion phase was characterized by magnetic flux accumulation.
6. The properties of the near‐Earth flows and dipolarizations were in general agreement with the observations by the GOES satellites at the geosynchronous orbit and MMS, located at ∼8.2 *R*_E_ radial distance. In particular, the strength of flows and dipolarizations were within a factor of 2 of those observed. Moreover, if the dipolarization was overestimated, the flow was underestimated, suggesting an even better agreement in the magnetic flux transport. Due to sporadic nature of BBFs/DFs, we do not expect the simulation to reproduce ideally even multipoint measurements. Thus, the above results serve as a strong confirmation of the validity of our results.
7. The simulations reproduced many previously reported observational signatures of BBFs and DFs in the near‐Earth region. In particular, we have demonstrated the existence of oscillating flux tubes that overshoot their equilibrium position and rebound tailward multiple times. The existence of strong vortical tailward flows on the flanks of the intruding BBF has also been demonstrated.
8. Spacecraft located away from the intruding BBF can still observe significant disturbances due to it. In particular, if the spacecraft is located away from the equatorial plane, it can observe a complex mixture of reconnection, rebound, and dipolarization signatures, resulting in plasma sheet expansion, thinning, and expansion again. This leads, in turn, to multiple spacecraft excursions from the lobe into the plasma sheet and back. Furthermore, BBFs disturb the plasma and magnetic field ahead and on the side of them in a region significantly exceeding their azimuthal size, which can be seen by spacecraft not necessarily located directly in the path of the penetrating structure.

Overall, this work demonstrates the power of high‐resolution simulations in providing deep physical insight, particularly, when used in conjunction with observations from multiple magnetospheric spacecraft. Given the high quality and sophistication of the underlying numerical algorithms running on modern computational resources, global models can now clearly capture many details of the real magnetospheric behavior, including processes occurring at mesoscale such as BBFs and DFs. It is when such model results are cross‐examined with the corresponding observational signatures that robust answers to physical questions can be gleaned.

## Supporting information



Supporting Information S1

Movie S1

Movie S2

## References

[jgra55233-bib-0001] Angelopoulos, V. , Baumjohann, W. , Kennel, C. F. , Coroniti, F. V. , Kivelson, M. G. , Pellat, R. , & Paschmann, G. (1992). Bursty bulk flows in the inner central plasma sheet. Journal of Geophysical Research, 97(A4), 4027–4039. 10.1029/91JA02701

[jgra55233-bib-0002] Angelopoulos, V. , Coroniti, F. V. , Kennel, C. F. , Kivelson, M. G. , Walker, R. J. , Russell, C. T. , & Harris, T. (1996). Multipoint analysis of a bursty bulk flow event on April 11, 1985. Journal of Geophysical Research, 101, 4967–4989. 10.1029/95JA02722

[jgra55233-bib-0003] Angelopoulos, V. , Kennel, C. F. , Coroniti, F. V. , Pellat, R. , Kivelson, M. G. , Walker, R. J. , & Gosling, J. T. (1994). Statistical characteristics of bursty bulk flow events. Journal of Geophysical Research, 99, 21257 10.1029/94JA01263

[jgra55233-bib-0004] Apatenkov, S. V. , Sergeev, V. A. , Kubyshkina, M. V. , Nakamura, R. , Baumjohann, W. , Runov, A. , & Khotyaintsev, Y. (2007). Multi‐spacecraft observation of plasma dipolarization/injection in the inner magnetosphere. Annales Geophysicae, 25, 801–814. 10.5194/angeo-25-801-2007

[jgra55233-bib-0005] Baumjohann, W. , Hesse, M. , Kokubun, S. , Mukai, T. , Nagai, T. , & Petrukovich, A. A. (1999). Substorm dipolarization and recovery. Journal of Geophysical Research, 104, 24,995–25,000. 10.1029/1999JA900282

[jgra55233-bib-0006] Baumjohann, W. , Paschmann, G. , & Luehr, H. (1990). Characteristics of high‐speed ion flows in the plasma sheet. Journal of Geophysical Research, 95, 3801–3809. 10.1029/JA095iA04p03801

[jgra55233-bib-0007] Baumjohann, W. , Pellinen, R. J. , Opgenoorth, H. J. , & Nielsen, E. (1981). Joint two‐dimensional observations of ground magnetic and ionospheric electric fields associated with auroral zone currents—Current systems associated with local auroral break‐ups. Planetary and Space Science, 29, 431–435. 10.1016/0032-0633(81)90087-8

[jgra55233-bib-0008] Birn, J. , & Hesse, M. (2013). The substorm current wedge in MHD simulations. Journal of Geophysical Research: Space Physics, 118, 3364–3376. 10.1002/jgra.50187

[jgra55233-bib-0009] Birn, J. , & Hesse, M. (2014a). The substorm current wedge: Further insights from MHD simulations. Journal of Geophysical Research: Space Physics, 119, 3503–3513. 10.1002/2014JA019863 PMC4497475 26167439

[jgra55233-bib-0010] Birn, J. , & Hesse, M. (2014b). The substorm current wedge: Further insights from MHD simulations. Journal of Geophysical Research: Space Physics, 119, 3503–3513. 10.1002/2014JA019863 PMC4497475 26167439

[jgra55233-bib-0011] Birn, J. , Hesse, M. , Haerendel, G. , Baumjohann, W. , & Shiokawa, K. (1999). Flow braking and the substorm current wedge. Journal of Geophysical Research, 104(A), 19,895–19,904. 10.1029/1999JA900173

[jgra55233-bib-0012] Birn, J. , Liu, J. , Runov, A. , Kepko, L. , & Angelopoulos, V. (2019). On the contribution of dipolarizing flux bundles to the substorm current wedge and to flux and energy transport. Journal of Geophysical Research: Space Physics, 124, 5408–5420. 10.1029/2019JA026658

[jgra55233-bib-0013] Birn, J. , Nakamura, R. , Panov, E. V. , & Hesse, M. (2011). Bursty bulk flows and dipolarization in MHD simulations of magnetotail reconnection. Journal of Geophysical Research, 116, A01210 10.1029/2010JA016083

[jgra55233-bib-0014] Burch, J. L. , Moore, T. E. , Torbert, R. B. , & Giles, B. L. (2016). Magnetospheric Multiscale overview and science objectives. Space Science Reviews, 199, 5–21. 10.1007/s11214-015-0164-9

[jgra55233-bib-0015] Chen, C. X. , & Wolf, R. A. (1999). Theory of thin‐filament motion in Earth's magnetotail and its application to bursty bulk flows. Journal of Geophysical Research, 104, 14,613–14,626. 10.1029/1999JA900005

[jgra55233-bib-0016] Cramer, W. D. , Raeder, J. , Toffoletto, F. , Gilson, M. , & Hu, B. (2017). Plasma sheet injections into the inner magnetosphere: Two‐way coupled OpenGGCM‐RCM model results. Journal of Geophysical Research: Space Physics, 122, 5077–5091. 10.1002/2017JA024104

[jgra55233-bib-0017] Dubyagin, S. , Sergeev, V. , Apatenkov, S. , Angelopoulos, V. , Runov, A. , Nakamura, R. , & Larson, D. (2011). Can flow bursts penetrate into the inner magnetosphere? Geophysical Research Letters, 38, L08102 10.1029/2011GL047016

[jgra55233-bib-0018] Dungey, J. W. (1961). Interplanetary magnetic field and the auroral zones. Physical Review Letters, 6(2), 47.

[jgra55233-bib-0019] Erickson, G. M. , & Wolf, R. (1980). Is steady convection possible in the Earth's magnetotail? 7(11), 897–900.

[jgra55233-bib-0020] Fedder, J. A. , Slinker, S. P. , Lyon, J. G. , & Elphinstone, R. D. (1995). Global numerical simulation of the growth phase and the expansion onset for a substorm observed by Viking. Journal of Geophysical Research, 100, 19083 10.1029/95JA01524

[jgra55233-bib-0021] Forsyth, C. , Fazakerley, A. , Rae, I. , Watt, C. , Murphy, K. , & Wild, J. A. (2014). In situ spatiotemporal measurements of the detailed azimuthal substructure of the substorm current wedge. Journal of Geophysical Research: Space Physics, 119, 927–946. 10.1002/2013JA019302 26167439 PMC4497475

[jgra55233-bib-0022] Forsyth, C. , Rae, I. J. , Murphy, K. R. , Freeman, M. P. , Huang, C. L. , Spence, H. E. , & Watt, C. E. J. (2016). What effect do substorms have on the content of the radiation belts? Journal of Geophysical Research: Space Physics, 121, 6292–6306. 10.1002/2016JA022620 27656336 PMC5014235

[jgra55233-bib-0023] Gkioulidou, M. , Ohtani, S. , Mitchell, D. , Ukhorskiy, A. , Reeves, G. , Turner, D. , Gjerloev, J. W. , Nosé, M. , Koga, K. , Rodriguez, J. V. , & Lanzerotti, L. J. (2015). Spatial structure and temporal evolution of energetic particle injections in the inner magnetosphere during the 14 July 2013 substorm event. Journal of Geophysical Research: Space Physics, 120, 1924–1938. 10.1002/2014JA020872

[jgra55233-bib-0024] Gordeev, E. , Sergeev, V. , Merkin, V. , & Kuznetsova, M. (2017). On the origin of plasma sheet reconfiguration during the substorm growth phase. Geophysical Research Letters, 44, 8696–8702. 10.1002/2017GL074539

[jgra55233-bib-0025] Gordeev, E. , Sergeev, V. , Tsyganenko, N. , Kuznetsova, M. , Rastaetter, L. , Raeder, J. , & Wiltberger, M. (2017). The substorm cycle as reproduced by global MHD models. Space Weather, 15, 131–149. 10.1002/2016SW001495

[jgra55233-bib-0026] Hsieh, M. S. , & Otto, A. (2015). Thin current sheet formation in response to the loading and the depletion of magnetic flux during the substorm growth phase. Journal of Geophysical Research: Space Physics, 120, 4264–4278. 10.1002/2014JA020925

[jgra55233-bib-0027] Juusola, L. , Östgaard, N. , Tanskanen, E. , Partamies, N. , & Snekvik, K. (2011). Earthward plasma sheet flows during substorm phases. Journal of Geophysical Research, 116, A10228 10.1029/2011JA016852

[jgra55233-bib-0028] Kepko, L. , McPherron, R. L. , Amm, O. , Apatenkov, S. , Baumjohann, W. , Birn, J. , & Sergeev, V. (2015). Substorm current wedge revisited. Space Science Reviews, 190(1–4), 1–46. 10.1007/s11214-014-0124-9

[jgra55233-bib-0029] Lin, Y. , Wang, X. , Lu, S. , Perez, J. , & Lu, Q. (2014). Investigation of storm time magnetotail and ion injection using three‐dimensional global hybrid simulation. Journal of Geophysical Research: Space Physics, 119, 7413–7432. 10.1002/2014JA020005

[jgra55233-bib-0030] Liu, J. , Angelopoulos, V. , Chu, X. , Zhou, X. Z. , & Yue, C. (2015). Substorm current wedge composition by wedgelets. Geophysical Research Letters, 42, 1669–1676. 10.1002/2015GL063289

[jgra55233-bib-0031] Liu, J. , Angelopoulos, V. , Yao, Z. , Chu, X. , Zhou, X. Z. , & Runov, A. (2018). The current system of dipolarizing flux bundles and their role as wedgelets in the substorm current wedge. Electric Currents in Geospace and Beyond, 235, 323–337. 10.1002/9781119324522.ch19

[jgra55233-bib-0032] Liu, J. , Angelopoulos, V. , Zhang, X. J. , Turner, D. L. , Gabrielse, C. , Runov, A. , & Spence, H. (2016). Dipolarizing flux bundles in the CIS‐geosynchronous magnetosphere: Relationship between electric fields and energetic particle injections. Journal of Geophysical Research: Space Physics, 121, 1362–1376. 10.1002/2015JA021691

[jgra55233-bib-0033] Liu, J. , Angelopoulos, V. , Zhou, X. Z. , & Runov, A. (2014). Magnetic flux transport by dipolarizing flux bundles. Journal of Geophysical Research: Space Physics, 119, 909–926. 10.1002/2013JA019395

[jgra55233-bib-0034] Lotko, W. , Smith, R. H. , Zhang, B. , Ouellette, J. E. , Brambles, O. J. , & Lyon, J. G. (2014). Ionospheric control of magnetotail reconnection. Science, 345(6193), 184–187. 25013068 10.1126/science.1252907

[jgra55233-bib-0035] Lyon, J. G. , Fedder, J. A. , & Mobarry, C. M. (2004). The Lyon‐Fedder‐Mobarry (LFM) global MHD magnetospheric simulation code. Journal of Atmospheric and Solar: Terrestrial Physics, 66, 1333 10.1016/j.jastp.2004.03.020

[jgra55233-bib-0036] Malykhin, A. Y. , Grigorenko, E. E. , Kronberg, E. A. , Koleva, R. , Ganushkina, N. Y. , Kozak, L. , & Daly, P. W. (2018). Contrasting dynamics of electrons and protons in the near‐Earth plasma sheet during dipolarization. Annales Geophysicae, 36, 741–760. 10.5194/angeo-36-741-2018

[jgra55233-bib-0037] Mauk, B. H. , & McIlwain, C. E. (1974). Correlation of Kp with the substorm‐injected plasma boundary. Journal of Geophysical Research, 79, 3193–3196. 10.1029/JA079i022p03193

[jgra55233-bib-0038] McPherron, R. , Hsu, T. S. , Kissinger, J. , Chu, X. , & Angelopoulos, V. (2011). Characteristics of plasma flows at the inner edge of the plasma sheet. Journal of Geophysical Research, 116, A00I33 10.1029/2010JA015923

[jgra55233-bib-0039] Merkin, V. G. , Anderson, B. J. , Lyon, J. G. , Korth, H. , Wiltberger, M. , & Motoba, T. (2013). Global evolution of Birkeland currents on 10 min timescales: MHD simulations and observations. Journal of Geophysical Research: Space Physics, 118, 4977–4997. 10.1002/jgra.50466

[jgra55233-bib-0040] Merkin, V. G. , & Lyon, J. G. (2010). Effects of the low‐latitude ionospheric boundary condition on the global magnetosphere. Journal of Geophysical Research, 115, A10202 10.1029/2010JA015461

[jgra55233-bib-0041] Merkin, V. G. , Lyon, J. G. , & Claudepierre, S. G. (2013). Kelvin‐Helmholtz instability of the magnetospheric boundary in a three‐dimensional global MHD simulation during northward IMF conditions. Journal of Geophysical Research: Space Physics, 118, 5478–5496. 10.1002/jgra.50520

[jgra55233-bib-0042] Moore, T. E. , Arnoldy, R. L. , Feynman, J. , & Hardy, D. A. (1981). Propagating substorm injection fronts. Journal of Geophysical Research, 86, 6713–6726. 10.1029/JA086iA08p06713

[jgra55233-bib-0043] Motoba, T. , Ohtani, S. , Gkioulidou, M. , Ukhorskiy, A. , Mitchell, D. , Takahashi, K. , & Wygant, J. (2018). Response of different ion species to local magnetic dipolarization inside geosynchronous orbit. Journal of Geophysical Research: Space Physics, 123, 5420–5434. 10.1029/2018JA025557

[jgra55233-bib-0044] Nakamura, R. , Baker, D. N. , Yamamoto, T. , Belian, R. D. , Bering, E. A. III , Benbrook, J. R. , & Theall, J. R. (1994). Particle and field signatures during pseudobreakup and major expansion onset. Journal of Geophysical Research, 99, 207–221. 10.1029/93JA02207

[jgra55233-bib-0045] Nakamura, R. , Baumjohann, W. , Klecker, B. , Bogdanova, Y. , Balogh, A. , Réme, H. , Bosqued, J. M. , Dandouras, I. , Sauvaud, J. A. , Glassmeier, K.‐H. , Kistler, L. , Mouikis, C. , Zhang, T. L. , Eichelberger, H. , & Runov, A. (2002). Motion of the dipolarization front during a flow burst event observed by Cluster. Geophysical research letters, 29(20), 3–1.

[jgra55233-bib-0046] Nakamura, R. , Baumjohann, W. , Mouikis, C. , Kistler, L. M. , Runov, A. , Volwerk, M. , & Balogh, A. (2004). Spatial scale of high‐speed flows in the plasma sheet observed by Cluster. Geophysical Research Letters, 31, L09804 10.1029/2004GL019558

[jgra55233-bib-0047] Ohtani, S. , Miyashita, Y. , Singer, H. , & Mukai, T. (2009). Tailward flows with positive Bz in the near‐Earth plasma sheet. Journal of Geophysical Research, 114, A06218 10.1029/2009JA014159

[jgra55233-bib-0048] Ohtani, S. , Motoba, T. , Gkioulidou, M. , Takahashi, K. , & Singer, H. (2018). Spatial development of the dipolarization region in the inner magnetosphere. Journal of Geophysical Research: Space Physics, 123, 5452–5463. https://doi.org/0.1029/2018JA025443

[jgra55233-bib-0049] Ohtani, S. , & Mukai, T. (2006). Plasma sheet expansion: Statistical characteristics. Journal of Geophysical Research, 111, A05206 10.1029/2005JA011547

[jgra55233-bib-0050] Ohtani, S. , Shay, M. A. , & Mukai, T. (2004). Temporal structure of the fast convective flow in the plasma sheet: Comparison between observations and two‐fluid simulations. Journal of Geophysical Research, 109, 3210 10.1029/2003JA010002

[jgra55233-bib-0051] Ohtani, S. , Singer, H. , & Mukai, T. (2006). Effects of the fast plasma sheet flow on the geosynchronous magnetic configuration: Geotail and GOES coordinated study. Journal of Geophysical Research, 111, A01204 10.1029/2005JA011383

[jgra55233-bib-0052] Ohtani, S. , Takahashi, K. , Zanetti, L. , Potemra, T. , McEntire, R. , & Iijima, T. (1992). Initial signatures of magnetic field and energetic particle fluxes at tail reconfiguration: Explosive growth phase. Journal of Geophysical Research, 97(A12), 19311–19324.

[jgra55233-bib-0053] Palin, L. , Opgenoorth, H. J. , Ågren, K. , Zivkovic, T. , Sergeev, V. A. , Kubyshkina, M. V. , & Nakamura, R. (2016). Modulation of the substorm current wedge by bursty bulk flows: 8 September 2002—Revisited. Journal of Geophysical Research: Space Physics, 121, 4466–4482. 10.1002/2015JA022262

[jgra55233-bib-0054] Panov, E. V. , Baumjohann, W. , Nakamura, R. , Weygand, J. M. , Giles, B. L. , Russell, C. T. , & Kubyshkina, M. V. (2019). Continent‐wide R1/R2 current system and ohmic losses by broad dipolarization‐injection fronts. Journal of Geophysical Research: Space Physics, 124, 4064–4082. 10.1029/2019JA026521

[jgra55233-bib-0055] Panov, E. V. , Baumjohann, W. , Wolf, R. A. , Nakamura, R. , Angelopoulos, V. , Weygand, J. M. , & Kubyshkina, M. V. (2016). Magnetotail energy dissipation during an auroral substorm. Nature Physics, 12, 1158–1163. 10.1038/nphys3879 27917231 PMC5131847

[jgra55233-bib-0056] Panov, E. V. , Nakamura, R. , Baumjohann, W. , Angelopoulos, V. , Petrukovich, A. A. , Retinò, A. , & Larson, D. (2010). Multiple overshoot and rebound of a bursty bulk flow. Geophysical Research Letters, 37, L08103 10.1029/2009GL041971

[jgra55233-bib-0057] Panov, E. V. , Nakamura, R. , Baumjohann, W. , Sergeev, V. A. , Petrukovich, A. A. , Angelopoulos, V. , & Larson, D. (2010). Plasma sheet thickness during a bursty bulk flow reversal. Journal of Geophysical Research, 115, A05213 10.1029/2009JA014743

[jgra55233-bib-0058] Petrukovich, A. (2011). Origins of plasma sheet By. Journal of Geophysical Research, 116, A07217 10.1029/2010JA016386

[jgra55233-bib-0059] Pollock, C. , Moore, T. , Jacques, A. , Burch, J. , Gliese, U. , Saito, Y. , & Zeuch, M. (2016). Fast Plasma Investigation for Magnetospheric Multiscale. Space Science Reviews, 199, 331–406. 10.1007/s11214-016-0245-4

[jgra55233-bib-0060] Pontius, D. Jr , & Wolf, R. (1990). Transient flux tubes in the terrestrial magnetosphere. Geophysical research letters, 17(1), 49–52.

[jgra55233-bib-0061] Reeves, G. D. , Fritz, T. A. , Cayton, T. E. , & Belian, R. D. (1990). Multi‐satellite measurements of the substorm injection region. Geophysical Research Letters, 17, 2015–2018. 10.1029/GL017i011p02015

[jgra55233-bib-0062] Rostoker, G. (1991). Some observational constraints for substorm models. Magnetospheric substorms, 64, 61–72.

[jgra55233-bib-0063] Runov, A. , Angelopoulos, V. , Sitnov, M. I. , Sergeev, V. A. , Bonnell, J. , McFadden, J. P. , & Auster, U. (2009). THEMIS observations of an earthward‐propagating dipolarization front. Geophysical Research Letters, 36, 14106 10.1029/2009GL038980

[jgra55233-bib-0064] Sandhu, J. K. , Rae, I. J. , Freeman, M. P. , Forsyth, C. , Gkioulidou, M. , Reeves, G. D. , & Lam, M. M. (2018). Energization of the ring current by substorms. Journal of Geophysical Research: Space Physics, 123, 8131–8148. 10.1029/2018JA025766 30775195 PMC6360953

[jgra55233-bib-0065] Sergeev, V. , Angelopoulos, V. , Apatenkov, S. , Bonnell, J. , Ergun, R. , Nakamura, R. , & Runov, A. (2009). Kinetic structure of the sharp injection/dipolarization front in the flow‐braking region. Geophysical Research Letters, 36, L21105 10.1029/2009GL040658

[jgra55233-bib-0066] Sergeev, V. A. , Angelopoulos, V. , Gosling, J. T. , Cattell, C. A. , & Russell, C. T. (1996). Detection of localized, plasma‐depleted flux tubes or bubbles in the midtail plasma sheet. Journal of Geophysical Research, 101, 10,817–10,826. 10.1029/96JA00460

[jgra55233-bib-0067] Sergeev, V. , Chernyaev, I. , Dubyagin, S. , Miyashita, Y. , Angelopoulos, V. , Boakes, P. , & Henderson, M. (2012). Energetic particle injections to geostationary orbit: Relationship to flow bursts and magnetospheric state. Journal of Geophysical Research, 117, A10207 10.1029/2012JA017773

[jgra55233-bib-0068] Sergeev, V. A. , Sauvaud, J. A. , Popescu, D. , Kovrazhkin, R. A. , Liou, K. , Newell, P. T. , & Reeves, G. D. (2000). Multiple‐spacecraft observation of a narrow transient plasma jet in the Earth's plasma sheet. Geophysical Research Letters, 27, 851–854. 10.1029/1999GL010729

[jgra55233-bib-0069] Shiokawa, K. , Baumjohann, W. , & Haerendel, G. (1997). Braking of high‐speed flows in the near‐Earth tail. Geophysical Research Letters, 24, 1179–1182. 10.1029/97GL01062

[jgra55233-bib-0070] Sitnov, M. , Birn, J. , Ferdousi, B. , Gordeev, E. , Khotyaintsev, Y. , Merkin, V. , Motoba, T. , Otto, A. , Panov, E. , Pritchett, P. , Pucci, F. , Raeder, J. , Runov, A. , Sergeev, V. , Velli, M. , & Zhou, X. (2019). Explosive magnetotail activity. Space science reviews, 215(4), 31. 31178609 10.1007/s11214-019-0599-5 PMC6528807

[jgra55233-bib-0071] Sitnov, M. , Merkin, V. , Swisdak, M. , Motoba, T. , Buzulukova, N. , Moore, T. , & Ohtani, S. (2014). Magnetic reconnection, buoyancy, and flapping motions in magnetotail explosions. Journal of Geophysical Research: Space Physics, 119, 7151–7168. 10.1002/2014JA020205

[jgra55233-bib-0072] Sorathia, K. A. , Ukhorskiy, A. Y. , Merkin, V. G. , Fennell, J. F. , & Claudepierre, S. G. (2018). Modeling the depletion and recovery of the outer radiation belt during a geomagnetic storm: Combined MHD and test particle simulations. Journal of Geophysical Research: Space Physics, 123, 5590–5609. 10.1029/2018JA025506

[jgra55233-bib-0073] Takada, T. , Nakamura, R. , Baumjohann, W. , Asano, Y. , Volwerk, M. , Zhang, T. , & Carr, C. (2006). Do BBFs contribute to inner magnetosphere dipolarizations: Concurrent Cluster and Double Star observations. Geophysical research letters, 33, L21109 10.1029/2006GL027440

[jgra55233-bib-0074] Torbert, R. B. , Russell, C. T. , Magnes, W. , Ergun, R. E. , Lindqvist, P. A. , Le Contel, O. , & Lappalainen, K. (2016). The FIELDS instrument suite on MMS: Scientific objectives, measurements, and data products. Space Science Reviews, 199, 105–135. 10.1007/s11214-014-0109-8

[jgra55233-bib-0075] Turner, D. L. , Fennell, J. , Blake, J. , Claudepierre, S. , Clemmons, J. , Jaynes, A. , Leonard, T. , Baker, D. N. , Cohen, I. J. , Gkioulidou, M. , Ukhorskiy, A. Y. , Mauk, B. H. , Gabrielse, C. , Angelopoulos, V. , Strangeway, R. J. , Kletzing, C. A. , Le Contel, O. , Spence, H. E. , Torbert, R. B. , Burch, J. L. , & Reeves, G. D. (2017). Multipoint observations of energetic particle injections and substorm activity during a conjunction between Magnetospheric Multiscale (MMS) and Van Allen Probes. Journal of Geophysical Research: Space Physics, 122, 11,481–11,504. 10.1002/2017JA024554

[jgra55233-bib-0076] Ukhorskiy, A. Y. , Sorathia, K. A. , Merkin, V. G. , Sitnov, M. I. , Mitchell, D. G. , & Gkioulidou, M. (2018). Ion trapping and acceleration at dipolarization fronts: High‐resolution MHD and test‐particle simulations. Journal of Geophysical Research: Space Physics, 123, 5580–5589. 10.1029/2018JA025370

[jgra55233-bib-0077] Wiltberger, M. , Merkin, V. , Lyon, J. G. , & Ohtani, S. (2015). High‐resolution global magnetohydrodynamic simulation of bursty bulk flows. Journal of Geophysical Research: Space Physics, 120, 4555–4566. 10.1002/2015JA021080

[jgra55233-bib-0078] Wiltberger, M. , Weigel, R. S. , Lotko, W. , & Fedder, J. A. (2009). Modeling seasonal variations of auroral particle precipitation in a global‐scale magnetosphere‐ionosphere simulation. Journal of Geophysical Research, 114, A01204 10.1029/2008JA013108

[jgra55233-bib-0079] Wolf, R. A. , Chen, C. X. , & Toffoletto, F. R. (2012). Thin filament simulations for Earth's plasma sheet: Interchange oscillations. Journal of Geophysical Research, 117, A02215 10.1029/2011JA016971

[jgra55233-bib-0080] Yang, J. , Toffoletto, F. , Wolf, R. , & Sazykin, S. (2011). RCM‐E simulation of ion acceleration during an idealized plasma sheet bubble injection. Journal of Geophysical Research, 116, A05207 10.1029/2010JA016346

[jgra55233-bib-0081] Yang, J. , Toffoletto, F. R. , Wolf, R. A. , & Sazykin, S. (2015). On the contribution of plasma sheet bubbles to the storm time ring current. Journal of Geophysical Research: Space Physics, 120, 7416–7432. 10.1002/2015JA021398

[jgra55233-bib-0281] Zhang, B. , Sorathia, K. A. , Lyon, J. G. , Merkin, V. G. , Garretson, J. S. , & Wiltberger, M. (2019). GAMERA: A three‐dimensional finite‐volume MHD solver for non‐orthogonal curvilinear geometries. The Astrophysical Journal Supplement Series, 244(1), 20 10.3847/1538-4365/ab3a4c

