# Supplementary material for: Contribution of Bursty Bulk Flows to the Global Dipolarization of the Magnetotail During an Isolated Substorm
Source: J Geophys Res Space Phys. 2019 Nov 13;124(11):8647–68. doi: 10.1029/2019JA026872 (PMC7066714; doi:10.1029/2019JA026872)
Supplement: Supplementary file 1 — Supporting Information S1 [file JGRA-124-8647-s001.pdf]

Supporting Information for "Contribution of bursty bulk flows to the global dipolarization of the magnetotail during an isolated substorm"

V. G. Merkin[1], E. V. Panov[2], K. A. Sorathia[1], and A. Ukhorskiy[1]

[1]The Johns Hopkins University Applied Physics Laboratory, Laurel, MD, USA

[2]Space Research Institute, Austrian Academy of Sciences, Graz, Austria

Contents of this file

1. Figures S1 to S2
2. Movies S1 to S2

Additional Supporting Information (Files uploaded separately)

1. Captions for Movies S1 to S2

Introduction Here we include two animations providing an overview of the simulation and allowing the reader to see magnetospheric dynamics as they unravel in time. Additionally, we provide two figures that the reader might find useful in providing a broader context for analyzing and interpreting the results presented in the paper.

Corresponding author: V. G. Mekin, The Johns Hopkins University Applied Physics Laboratory, Laurel, MD 20723, USA. (slava.merkin@jhuapl.edu)

Movie S1. The movie is in the same format as Figures 2–4 in the paper. The SM equatorial plane is shown in each panel. Magnetospheric spacecraft are marked with blue (GOES-13), orange (GOES-14), green (GOES-15), and red (MMS-1) circles which indicate the spacecraft positions projected to the plane. The black contour in the upper left panel indicates the $B_Z = 0$ iso-contour.

Movie S2. The movie is in the same format as Figures 12 in the paper. It provides a version of Movie S1 zoomed-in on the central and near-Earth nightside magnetosphere but is otherwise identical.

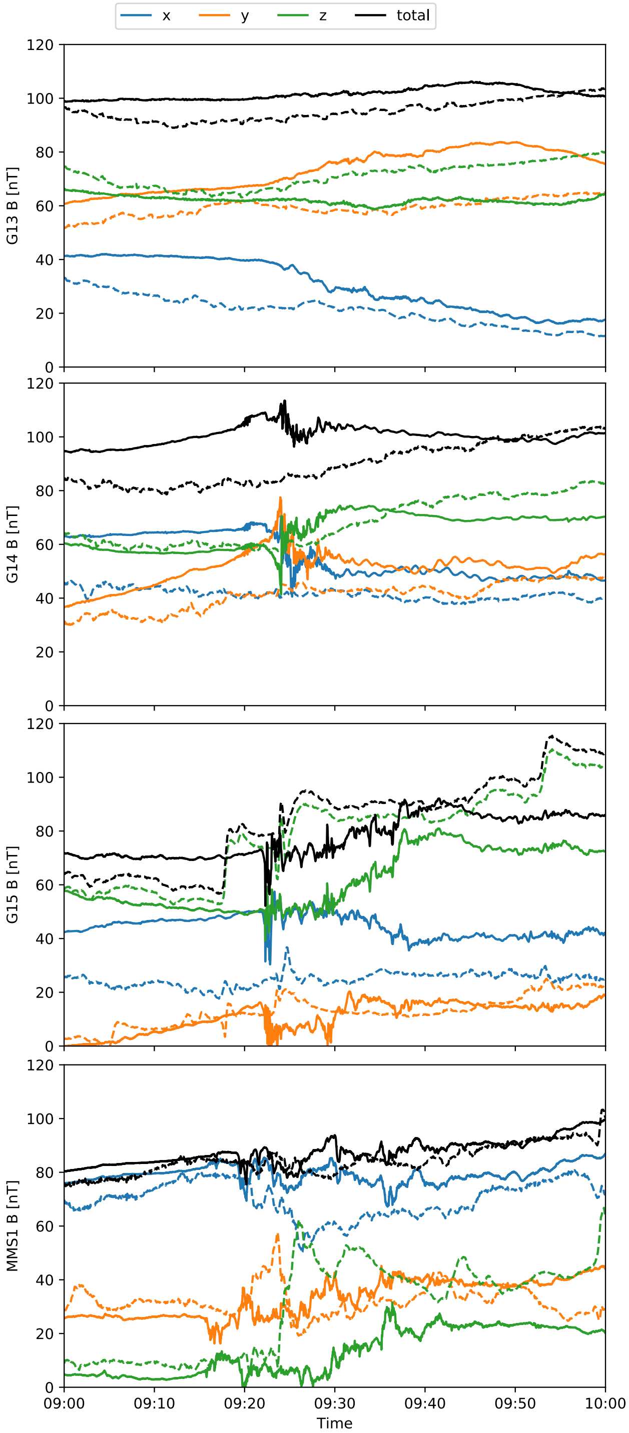

Figure S1. Observed and simulated magnetic field components in SM coordinates at the spacecraft indicated on the vertical axes. The simulated results are shown with dashed lines, while the actual observations are shown with solid lines. The color-coding for the different components as well as the magnetic field magnitude is indicated in the legend at the top. The sign of B_x is inverted for MMS-1 since it is located in the southern hemisphere. The corresponding spacecraft locations are shown in Figures 2, 3, 4, 11, and 12, and Figure S2 below.

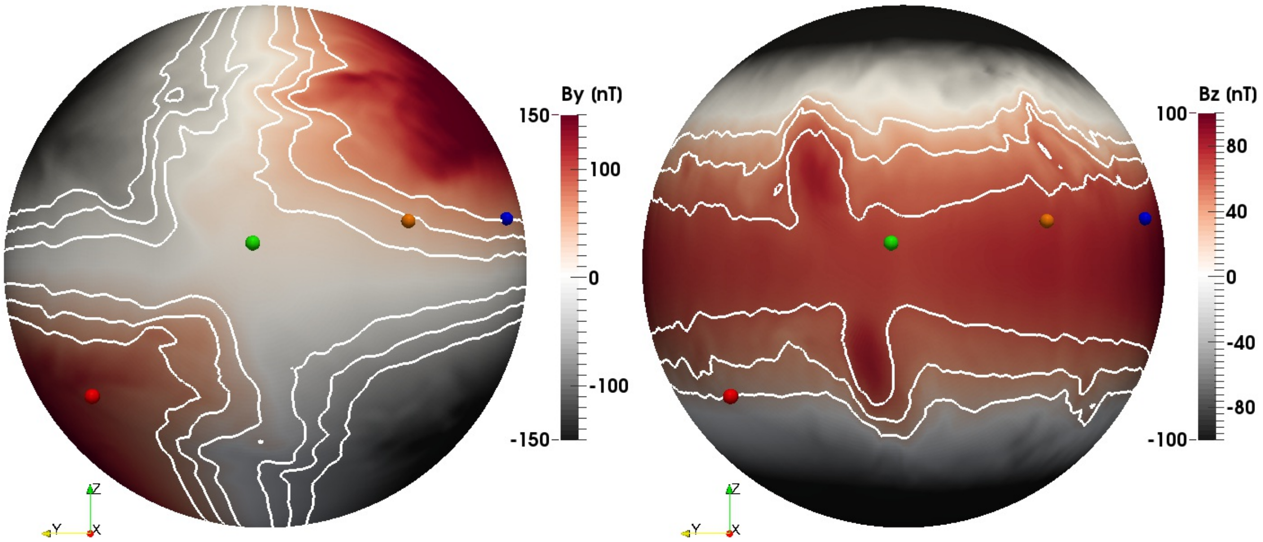

Figure S2. Same as Figure 11 in the paper but for the B_y and B_z magnetic field components at 09:15 UT. They are color-coded on a spherical slice through the simulation at the radial distance of 6.4 R_E. The view is from a vantage point on the SM x-axis down the tail looking toward Earth, i.e., dusk is to the left and dawn is to the right. The spacecraft are shown with small spherical glyphs color-coded the same way as in Figures 2, 3, 4, and 12: MMS-1 (red), GOES-15 (green), GOES-14 (orange), GOES-13 (blue). The white contours indicate $|B_{y,z}| = 20$, 40, and 60 nT.
